# Do economic effects of the anti-COVID-19 lockdowns in different regions interact through supply chains?

**DOI:** 10.1371/journal.pone.0255031

**Published:** 2021-07-30

**Authors:** Hiroyasu Inoue, Yohsuke Murase, Yasuyuki Todo

**Affiliations:** 1 Graduate School of Information Science, University of Hyogo, Kobe, Hyogo, Japan; 2 RIKEN Center for Computational Science, Kobe, Hyogo, Japan; 3 Graduate School of Economics, Waseda University, Tokyo, Japan; Sunway University, MALAYSIA

## Abstract

To prevent the spread of COVID-19, many cities, states, and countries have ‘locked down’, restricting economic activities in non-essential sectors. Such lockdowns have substantially shrunk production in most countries. This study examines how the economic effects of lockdowns in different regions interact through supply chains, which are a network of firms for production, by simulating an agent-based model of production using supply-chain data for 1.6 million firms in Japan. We further investigate how the complex network structure affects the interactions between lockdown regions, emphasising the role of upstreamness and loops by decomposing supply-chain flows into potential and circular flow components. We find that a region’s upstreamness, intensity of loops, and supplier substitutability in supply chains with other regions largely determine the economic effect of the lockdown in the region. In particular, when a region lifts its lockdown, its economic recovery substantially varies depending on whether it lifts the lockdown alone or together with another region closely linked through supply chains. These results indicate that the economic effect produced by exogenous shocks in a region can affect other regions and therefore this study proposes the need for inter-region policy coordination to reduce economic loss due to lockdowns.

## 1 Introduction

COVID-19, a novel coronavirus (SARS-CoV-2) disease, has been spreading worldwide. To prevent its spread, many cities, regions, and countries were or have been under lockdown, suppressing economic activities. On 18 April 2020, 158 countries out of 181 implemented measures that required temporary closure or work-from-home for some sectors in some or all cities. Although some countries later lifted their lockdowns, 95 countries remained under lockdown on 30 July 2020 [1].

Closing workplaces shrinks the economic output of regions under lockdown. The negative economic effect of a lockdown in one region may diffuse through supply chains, i.e., supplier-client relationships of firms, and to other regions that are not necessarily in a lockdown. When a firm is closed due to a lockdown strategy, its client firms located elsewhere would suffer decreased production due to the lack of supply of intermediate goods and services. Suppliers of the closed firms would also see reduced production because of a shortage of demand.

Many studies have empirically confirmed the propagation of economic shocks through supply chains, particularly shocks originating from natural disasters [2–7]. Some examine the diffusion of the effect of lockdowns because of COVID-19 on production across regions and countries and estimate the total effect using input–output (IO) linkages at the country-sector level [8–11] and supply chains at the firm level [12].

Several studies focusing on natural disasters [5, 6] examine how the network structure of supply chains affects the propagation of shocks. They find that scale-free property, non-substitutability of suppliers, and loops are major drivers of such propagation. However, the role of the network structure has not been fully examined in the context of the propagation of the lockdown effect. This issue should be of great interest from the perspective of network science for the following two reasons.

First, the literature on network interventions has investigated the types of individuals or groups in a network, such as those with high centrality, who should be targeted to promote (prevent) the diffusion of positive (negative) behaviours and outcomes [13, 14]. Similarly, we are interested in how the economic effect of imposing and lifting a lockdown in one region, an example of a network intervention, diffuses to other regions. Compared to existing research, this study is novel in many respects. For example, we consider interventions in a network of firms and their economic outcomes, while previous studies focus on the health behaviours and outcomes in human networks [15], with a few exceptions that examine economic outcomes in human networks [16]. In addition, because a lockdown is usually imposed in a city, state, or country, the scale of interventions is large. Firms targeted by such interventions are exogenously determined by geography, and thus we should assess the network characteristics of exogenously grouped nodes, rather than the endogenously connected ones identified by network centrality [13, 17] or community detection algorithms [18].

Second, at any point during the spread of COVID-19, some regions imposed a lockdown, while others remained open. Therefore, when we evaluate the lockdown strategy of a region, the interactions between the strategies of different regions need to be considered. In other words, the economic effect of a lockdown in a region depends on whether other regions connected to it through supply chains are similarly locked down. For example, Sweden did not impose a strict lockdown, unlike other European countries. However, it still expects a 4.5% reduction in gross domestic product (GDP) in 2020, a decline comparable to that in neighbouring countries that did impose a lockdown, possibly because of its close economic ties with its neighbours [19]. Motivated by the Swedish experience, this study examines the network structure between regions—an aspect that is usually ignored in the literature on network interventions—and discusses the need for policy coordination among regions depending on their network characteristics. Some studies call for inter-regional and international policy coordination in the presence of spillover effects in the context of health, environment, and macroeconomics [20, 21], but they do not explicitly incorporate the network structure.

The present study fills the above gaps in research on network interventions and regional interactions. We conduct a simulation analysis by applying actual supply-chain data of 1.6 million firms and their experiences of the lockdowns in Japan to an agent-based model of production. Specifically, we analyse the network characteristics of a prefecture in Japan that led to greater economic recovery by lifting its lockdown when all other prefectures remained locked down. In addition, to further highlight the interactions between regions, our simulation investigates how the characteristics of the supply-chain links between two prefectures affect their economic recovery when they simultaneously lift their lockdowns. One novelty of our study is to decompose supply-chain flows into potential and loop flow components and test the role of upstreamness (potential) in supply chains and intra- and inter-prefectural loops in diffusion.

## 2 Data

The data used in this study are taken from the Company Information Database and Company Linkage Database compiled by Tokyo Shoko Research (TSR), one of the largest credit research companies in Japan. The former database includes information about the attributes of each firm, including the location, industry, sales, and number of employees, and the latter includes the major customers and suppliers of each firm. Due to availability, we use the data on firm attributes and supply chains from 2016. The number of firms in the data is 1,668,567 and the number of supply-chain links is 5,943,073. Hence, our data identify the major supply chains of most firms in Japan, although they lack information about supply-chain links with foreign entities. Because the transaction value of each supply-chain tie is not available in the data, we estimate sales from a supplier to each of its customers and consumers using the total sales of the supplier and the 2015 Input-Output (IO) Tables for Japan [22]. In this estimation process, we drop firms without any sales information. Accordingly, the number of firms in our final analysis is 966,627 and the number of links is 3,544,343. Although the firms in the TSR data are classified into 1,460 industries according to the Japan Standard Industrial Classification [23], we simplify this into the 187 industries classified in the IO tables. S1 Appendix provides details on the data construction process.

In the supply-chain data described above, the degree, or the number of links, of firms follows a power-law distribution [5], as often found in the literature [24]. The average path length between firms, or the number of steps between them through supply chains, is 4.8, indicating a small-world network. Using the same dataset, previous studies [5, 25] find that 46–48% of firms are included in the giant strongly connected component (GSCC), in which all firms are indirectly connected to each other through supply chains. The large size of the GSCC clearly shows that the network has a significant number of cycles unlike the common image of a layered or tree-like supply-chain structure.

## 3 Methods

### 3.1 Model

Agent-based models that incorporate the interactions of agents through networks have been widely used in the social sciences [26–28]. Following the literature, we employ the dynamic agent-based model of Inoue and Todo [5, 6], an extension of Hallegatte’s [29] model, which assumes that supply chains are at the firm level. In the model, each firm utilises the inputs purchased from other firms to produce an output and sells it to other firms and consumers. Firms in the same industry are assumed to produce the same output. Supply chains are predetermined, and do not change over time in the following two respects. First, each firm utilises a firm-specific set of input varieties and does not change the input set over time. Second, each firm is linked with fixed suppliers and customers and cannot be linked with any new firm over time, even after a supply-chain disruption. Accordingly, our analysis focuses on short-term changes in production. Furthermore, we assume that each firm keeps inventories of each input at a level randomly determined from the Poisson distribution. Following Inoue and Todo [5], in which parameter values are calibrated from the case of the Great East Japan earthquake, we assume that firms aim to keep inventories for 10 days of production on average (see S2 Appendix for the details).

When a restriction is imposed on a firm’s production, both its upstream and downstream of the firm are affected. On the one hand, the firm’s demand for parts and components from its suppliers immediately declines, and thus suppliers have to shrink their production. Because demand for the products of suppliers’ suppliers also declines, the negative effect of the restriction propagates upstream. On the other hand, the supply of products from the directly restricted firm to its customer firms declines. Therefore, one way for customer firms to maintain the current level of production is to use their inventories of inputs. Alternatively, customers can procure inputs from other suppliers in the same industry that were already connected before the restriction, provided these suppliers have additional production capacity. If the inventories and inputs from substitute suppliers are insufficient, customers have to shrink their production because of a shortage of inputs. Accordingly, the effect of the restriction propagates downstream through supply chains. Such downstream propagation is likely to be slower than upstream propagation because of the inventory buffer and input substitution.

### 3.2 Lockdowns in Japan

In Japan, lockdown strategies were implemented at the prefecture level under the state of emergency [30] first declared on 7 April, 2020 in seven prefectures with a large number of confirmed COVID-19 cases. Because populated regions tended to be affected more and earlier, these seven prefectures are industrial clusters in Japan, including Tokyo, Osaka, Fukuoka, and their neighbouring prefectures. The state of emergency was expanded to all 47 prefectures on 16 April. The state of emergency was lifted for 39 prefectures on 14 May and for an additional three on 21 May; it was lifted for the remaining five prefectures on 25 May. (The summary of the timeline of the lockdowns in different prefectures can be found in Fig A.3 of [31]).

Although the national government declared a state of emergency, the extent to which the restrictions were imposed was determined by each prefecture’s government. Therefore, the level of lockdown in each prefecture may have varied. Although all prefectures were in the state of emergency from 16 April to 14 May, prefectures with larger numbers of confirmed COVID-19 cases, such as the seven prefectures in which a state of emergency was first declared, requested more stringent restrictions than others. The national or prefectural government can only request closing workplaces, staying at home, and social distancing rather than enforcing these actions through legal enforcement or punishment. However, strong social pressure in Japan led people and businesses to voluntarily restrict their activities to a large extent. As a result, production activities including those in sectors not officially restricted shrunk substantially (Mainichi Newspaper, 27 May 2020).

### 3.3 Simulation procedure

#### 3.3.1 Replication of the actual effect

In our simulation analysis, we first confirm whether our model and data can replicate the actual reduction in production caused by the lockdown in Japan during this state of emergency. Because we cannot observe the extent to which each firm reduces its production capacity by obeying government requests, the rate of reduction in production capacity for each sector assumed in our simulation analysis depends on its characteristics. As the reduction rate, particularly during the lockdowns in Japan is not available, we follow the literature that defines the reduction rate in general settings. Specifically, the rate of reduction in a sector is the product of the level of reduction determined by the degree of exposure to the virus given by [9] and the share of workers who cannot work from home given by [8]. For example, in lifeline/essential sectors such as utilities, health, and transport, the rate of reduction is assumed to be zero; in other words, the production capacity in these sectors does not change during a lockdown. In sectors in which it is assumed that exposure to the virus is low (50%) and 13.4% of workers can work from home, such as the agriculture and fishery sectors, the rate of reduction is 43.3% (= 0.5 × (1 − 0.134)). Sectors with ordinary exposure (100%) and 47.5% of workers were working from home, such as the retail and wholesale sectors, show a reduction in production capacity by 52.5% (= 1 × (1 − 0.475)). See S1 Table for the rate of reduction of each sector.

After the lockdown in a prefecture is lifted, all the firms in that prefecture immediately return to their pre-lockdown production capacity. Moreover, we assume that inventories do not decay over time: inventories stocked before the lockdown can be fully utilised after the lockdown is lifted. The results given below are an averaged of over 30 Monte Carlo runs.

#### 3.3.2 Interactions among regions

After checking the accuracy of our simulation model, we examine how changing the restriction level of the lockdown in a region affects production in another region. For this purpose, we experiment with different sets of sector-specific rates of reduction in production capacity by multiplying the benchmark rates of reduction defined above by a multiplier such as 0.4 or 0.8. For example, when the benchmark rate of reduction in a sector is 52.5%, as in the case of the iron and other metal product sectors, and the multiplier is 0.4, we alternatively assume a rate of reduction of 21.0%.

Moreover, we assume that the rates of reduction can vary among prefectures, because each prefecture can determine its own level of restrictions under the state of emergency (Section 3.2). In practice, the restrictions requested by the prefectural governments were tougher and people were more obedient to the requests in the seven prefectures in which the state of emergency was first declared because of the larger COVID-19 caseloads than in other prefectures. Accordingly, we run the same simulation assuming different rates of reduction for the two types of prefectures, defined as more and less restricted groups, to investigate how different rates of reduction in one group affect production in the other.

#### 3.3.3 Lifting lockdown in only one region

In practice, some prefectures lifted their lockdowns earlier than others (Section 3.2). Although this may have led to the recovery of value added production, or gross regional product (GRP), the extent of such a recovery should have been affected by the links between firms in the prefecture and others still under lockdown. To highlight this network effect, we simulate what would happen to the GRP of a prefecture if it lifted its lockdown while all others were still imposing lockdowns. Next, we investigate what network characteristics of each prefecture determine the recovery from lockdown, measured by the ratio of the increase in the GRP of the prefecture by lifting its lockdown to the reduction in its GRP because of the lockdown of all prefectures.

In particular, we focus on four types of network characteristics. First, when a prefecture is more isolated from others in the supply-chain network, the effect of others’ lockdowns should be smaller. We measure the level of isolation using the number of links within the prefecture relative to the total degree of firms (total number of links from and to firms) in the prefecture.

Second, an alternative and more interesting measure of isolation is the intensity of loops in supply chains. Although supply chains usually flow from suppliers of materials to those of parts and components and then to assemblers, some suppliers use final products such as machinery and computers as inputs. This results in many complex loops in supply chains [32], in which negative shocks circulate and can become aggravated [5]. Such loops in a network are found to generate instability in the system dynamics literature [33] and more recently in the context of supply chains [34]. In the case of lifting the lockdown in only one prefecture, the loops within that prefecture may magnify its recovery because of the circulation of positive effects in the loops.

**Fig 1:**
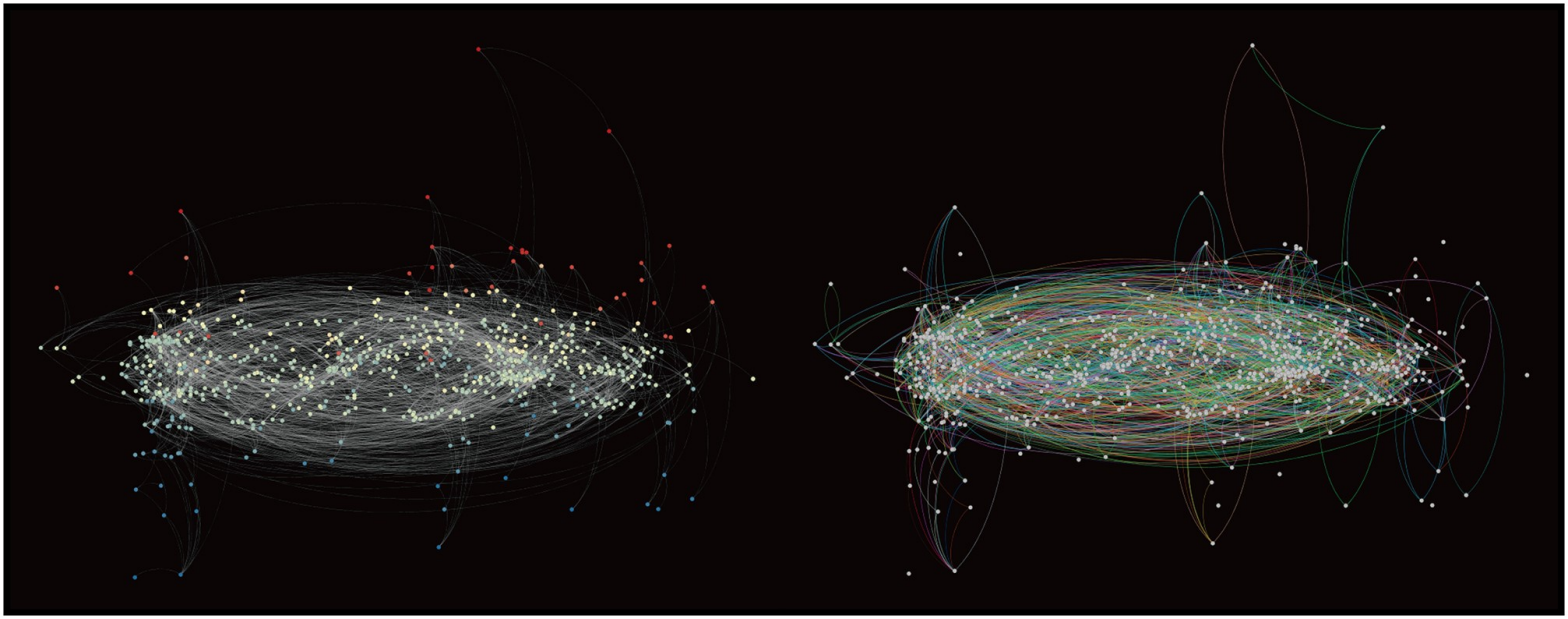
Visualisation of supply chains for top 1,000 firms in terms of sales. Each dot indicates a firm. Firms with a higher Helmholtz–Hodge (HH) potential are located more upward in both panels. In the left panel, the grey lines illustrate the potential flows computed from the HHD. The red and blue node colours represent higher and lower HH potentials, respectively. The right panel shows loop flows computed from HHD, while the different colours represent different cycles.

To measure the intensity of the loops in the supply chains within a prefecture, we apply the Helmholtz–Hodge decomposition (HHD) to all the flows in the network. We then decompose each directed link from firm *i* to firm *j*, *F*_*ij*_, into a potential (or gradient) flow component, $F_{ij}^{\left(p\right)}$, and a loop (or circular) flow component, $F_{ij}^{\left(c\right)}$ [35]. S1 Fig explains the details of the HHD. Fig 1 illustrates potential and loop flows of top 1,000 firms in terms of sales. In particular, the right panel identifies a number of loops in supply chains. Then, our measure of the intensity of the loops for prefecture *a* is the ratio of the total loop flows within the prefecture $\sum_{i,j\in a}F_{ij}^{\left(c\right)}$ to the total degree of all the firms in the prefecture denoted by *F*_*a*_.

Third, we pay attention to the upstreamness of firms in supply chains. Theoretically, upstream firms are affected by supply-chain disruptions through a lack of demand, whereas downstream firms are affected through a lack of supply. However, the effect of upstream and downstream links can differ in size. A recent sectoral analysis [36] finds that the profits of more upstream sectors in global value chains are substantially lower than those of more downstream sectors, implying that negative economic shocks propagate upstream more than downstream. To clarify the possible effect of upstreamness, we define the upstream position of each firm *i* in supply chains by its Helmholtz–Hodge (HH) potential, *φ*_*i*_ computed from the HHD. In other words, the hierarchical position of a firm can be consistently defined by focusing on gradient flows, in other words, all flows less loop flows. The HH potential is higher when the firm is located in a more upstream position. In practice, it is generally higher for firms in the mining, manufacturing, and information and communication sectors, while lower for those in the wholesale, retail, finance, healthcare, and accommodation and food service sectors [32]. We average the HH potential over the firms in each prefecture to measure the upstreamness of the prefecture in supply chains. The visualization on the map can be found in Fig B.2 of [31].

Our measure of upstreamness based on the HH potential, is conceptually similar to the upstreamness measures developed and widely used in the literature on international trade [37–41] in that both measure the hierarchical position in supply chains. However, a clear difference between the two types of measures is that ours is based on firm-level data while others are based on sector-level IO tables. Therefore, our measure can incorporate firm-level heterogeneity within the same sector that is ignored in others. In addition, our measure is defined by gradient flows in supply chains that are constructed by eliminating loop flows from all flows. Although many loops at the firm level are found in supply chains, even within the industry [32], upstream measures based on IO tables do not incorporate such loops. For these reasons, we will rely on our upstreamness measures at the firm level, and not on existing measures at the sector level.

Finally, even when the supply of parts and components from other prefectures is shut down because of their lockdowns, the negative effect can be mitigated if suppliers can be replaced by those in the prefecture lifting its lockdown. Existing studies [2, 5] have found that input substitutability can largely mitigate the propagation of negative economic shocks through supply chains. By assumption, suppliers of firms in prefecture *a* that are in other prefectures currently under lockdown can be replaced by suppliers in prefecture *a* that are in the same industry and already connected. To measure the degree of supplier substitutability for prefecture *a*, we divide the number of the latter suppliers by the number of the former.

#### 3.3.4 Lifting lockdowns in two regions simultaneously

In practice, each prefecture government determined the restriction level of its lockdown after observing the spread of COVID-19 in its prefecture and typically ignored the economic interactions with other prefectures through supply chains. This may have led to the misevaluation of the economic effect of lockdown. To emphasise the role of the interactions between prefectures with regard to the economic effects of lockdown, our simulations analyse the economic effect of lifting the lockdown on a prefecture’s GRP when another prefecture lifts its lockdown simultaneously. We define a relative measure of recovery using the ratio of the increase in the GRP of prefecture *a* when it lifts its lockdown, together with prefecture *b* ($\Delta GRP_{a}^{ab}$) to its increase when it lifts its lockdown alone ($\Delta GRP_{a}^{a}$).

Presumably, the characteristics of the links between the two prefectures largely affect their recovery. Expanding the case of lifting the lockdown in only one prefecture described just above, we are particularly interested in the following variables. First, we define the intensity of the directional links from prefectures *a* to *b* and from *b* to *a* by

$$\begin{array}{c} Link_{ab}\equiv \sum_{i\in a,j\in b}F_{ij}/F_{a} \end{array}$$ (1)

and

$$\begin{array}{c} Link_{ba}\equiv \sum_{i\in a,j\in b}F_{ji}/F_{a}, \end{array}$$ (2)

respectively, where *F*_*a*_ is the total degree of firms in prefecture *a*, as defined before. Second, we focus on potential flows using the HHD as above and define the intensity of potential flows from prefectures *a* to *b* and from *b* to *a* by

$$\begin{array}{c} Pot_{ab}\equiv \sum_{i\in a,j\in b}F_{ij}^{\left(p\right)}/F_{a} \end{array}$$ (3)

and

$$\begin{array}{c} Pot_{ba}\equiv \sum_{i\in a,j\in b}F_{ji}^{\left(p\right)}/F_{a}, \end{array}$$ (4)

respectively. Third, the intensity of the loops between prefectures *a* and *b* is given by

$$\begin{array}{c} Loop_{ab}\equiv \sum_{i\in a,j\in b}F_{ij}^{\left(c\right)}/F_{a}. \end{array}$$ (5)

S2 Appendix describes how to calculate *Pot*_*ab*_, *Pot*_*ba*_, and *Loop*_*ab*_ using a simple example.

Finally, when suppliers of firms in prefecture *a* are located outside prefectures *a* and *b* and thus are locked down, they can be replaced by suppliers in the same industry in prefecture *b* that are already connected with firms in prefecture *a*. To measure the degree of this supplier substitutability, we divide the total number of the latter suppliers by the total number of the former. See S2 Appendix for the details.

## 4 Results

### 4.1 Simulation of the effect of the actual lockdown

**Fig 2:**
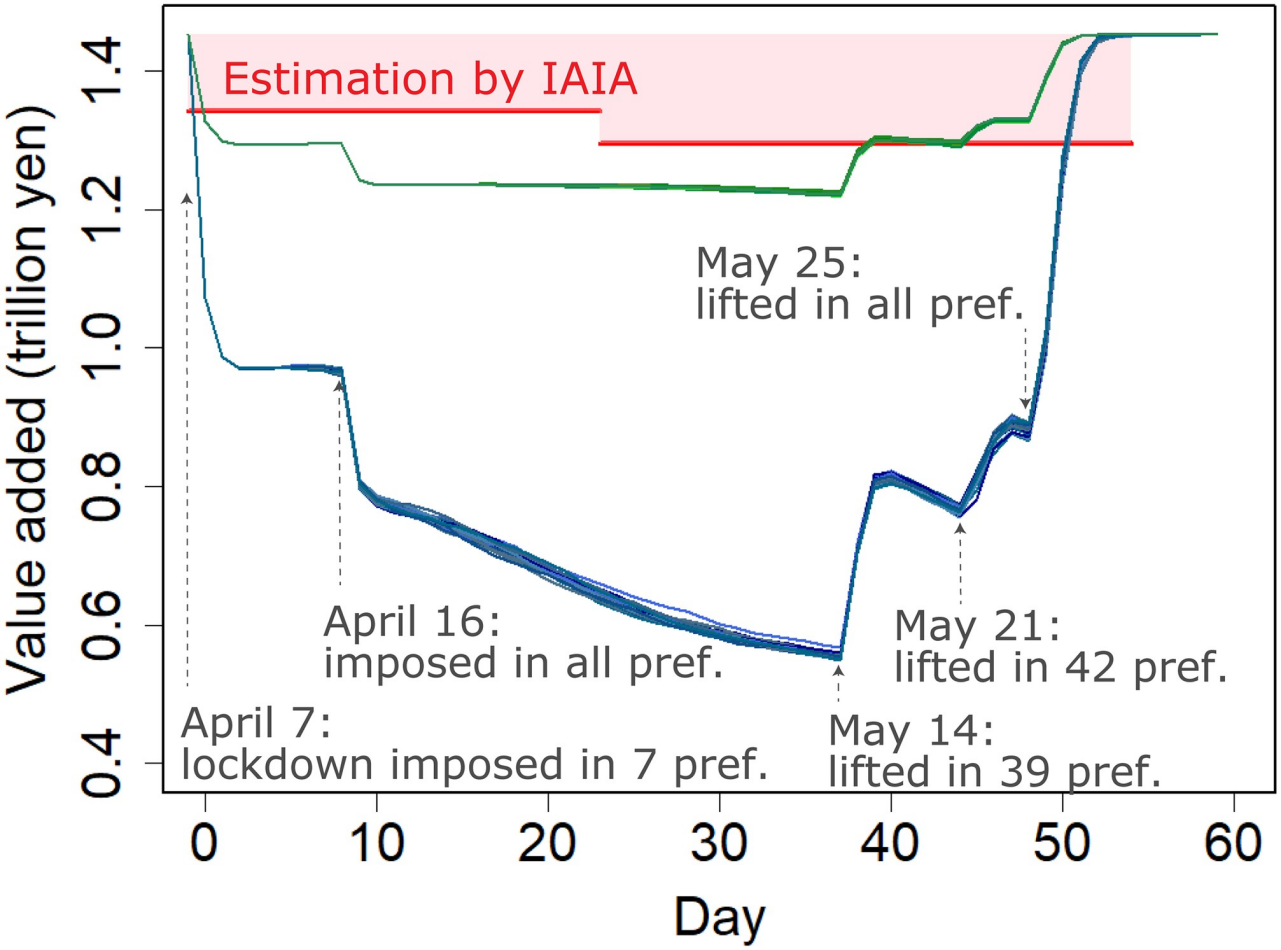
Simulations of value added (GDP) during the actual lockdown. The blue and green lines indicate the simulation results given the sector-specific rates of reduction in production capacity assumed in the literature [9, 36] and shown in S1 Table and the 26.7% of those rates to calibrate the actual production dynamics, respectively. Each line represents the daily GDP from one Monte Carlo run. The red segments indicate the daily GDP estimated from pre-lockdown GDP and the post-lockdown monthly Indices of All Industry Activity (IAIA) for April and May.

In Fig 2, the blue lines indicate the results of the 30 Monte Carlo runs conducted to estimate the effect of the actual lockdown in Japan given the sector-specific rates of reduction in production capacity assumed in the literature [9, 36] and shown in S1 Table. The horizontal axis indicates the number of days since the declaration of the state of emergency (7 April) and the vertical axis represents the total value added production, or GDP, of Japan on each day. See Section 3.2 for the sequence of the state of emergency across the country. Averaged over the 30 runs, the estimated loss in GDP is 35.0 trillion yen (3,280 billion U.S. dollars), or 6.60% of yearly GDP.

Without relying on our model and simulation, we also estimate the changes in daily GDP from pre-lockdown GDP and the post-lockdown monthly Indices of All Industry Activity (IAIA) [42]. The average daily GDP in April and May estimated from the IAIA is indicated by the red lines in Fig 2 (see S3 Appendix for the detailed procedures). The total loss of GDP estimated by the IAIA, or the pink area in Fig 2, is 7.52 trillion yen (1.44% of yearly GDP), 21.5% of the estimate from our simulations. Our simulation thus overestimates the loss of GDP from the lockdown, possibly because the assumed rates of reduction in production capacity due to the lockdown taken from the literature [8, 9] are larger than the actual rates in Japan. Therefore, we experiment with different rates of reduction in production capacity by multiplying the benchmark rates by a weight to calibrate changes in production. We find that a weight of 26.7% results in a close fit between our estimates and those from the IAIA, and indicate the results using green lines in Fig 2.

In either case (blue or green lines), the production loss rises during the lockdown. For example, the value added declined monotonically from days 9 to 37, when all prefectures were in a state of emergency, assuming a fixed rate of reduction in production capacity throughout the period. This is because the economic contraction in different regions interacted with each other through supply chains, and thus worsened over time. This worsening trend in GDP is consistent with GDP estimated using the IAIA.

Another notable finding from the simulation is that prefectures that were not locked down were heavily affected by those under lockdowns. The visualization on the map can be found in Fig 3 of [31]. In addition, a video presents a temporal and geographical visualisation of this. See S3 Appendix.

Moreover, because of the network effect, the earlier lifting of the lockdown in some prefectures does not result in a full recovery of production in these prefectures. Notably, when the lockdown was lifted in 39 prefectures on day 37 (14 May), the simulated GDP show a sharp recovery but drops again substantially a few days after the recovery. This drop occurred because the lockdown remained active in eight prefectures including the top two industrial clusters in Japan, greater Tokyo and greater Osaka. Although economic activities returned to normal in these 39 prefectures, their production did not recover monotonically but rather declined again because the major industrial clusters linked with them were still locked down. This finding points to the interactions of the economic effect of lockdown between regions through firm-level supply chains.

### 4.2 Interactions between lockdowns in different regions

**Fig 3:**
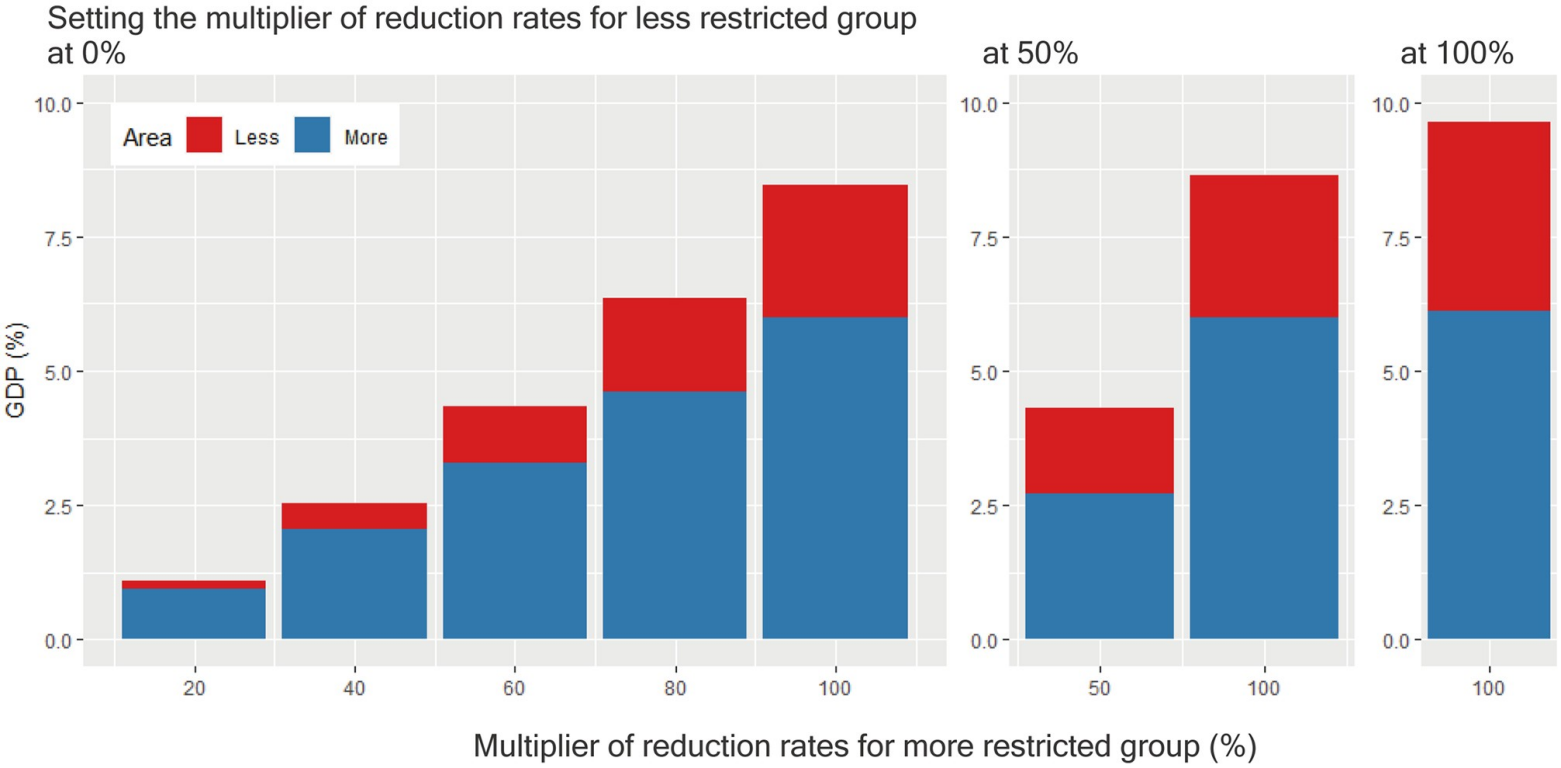
Loss in value added as a percentage of total value added (GDP) assuming different restriction levels of lockdown for 60 days between the more and less restricted groups. A restriction level is defined by a multiplier for the sector-specific benchmark rates of reduction in production capacity. For example, the left bar presents the result assuming a multiplier of 0% (i.e., no restriction) for the less restricted group and 20% for the more restricted group. The red and blue portions of each bar show the loss of value added in the less and more restricted groups, respectively, as a percentage of GDP.

Next, we experiment with simulations assuming different levels of restrictions, or different sets of multipliers for the sector-specific benchmark rates of reduction in production capacity, between the more and less restricted groups (Section 3.3). The more restricted group comprises the seven prefectures with a large number of COVID-19 cases, whereas the less restricted group includes the other 40 prefectures. The left, middle, and right panels of Fig 3 indicate the loss in GDP for different multipliers for the more restricted group when fixing the multiplier for the less restricted group at 0%, 50%, and 100%, respectively. Here, 100% corresponds to the rates of reduction shown in S1 Table and used in the previous subsection and 0% implies no restriction. In each bar, the blue and red portions indicate the loss of value added in the more and less restricted groups, respectively.

As shown, the total loss of GDP increases in the levels of restrictions in both groups. For example, the total production loss is 4.18% of GDP when the multiplier is 50% for both groups (the left bar in the middle panel), while it is larger, or 9.39%, when the multiplier is 100% for both (the right panel). More interestingly, the left panel shows that while the group with fewer restrictions imposes no restrictions, its value added decreases more (i.e., the red portion in Fig 3 increases) as the group with more restrictions imposes more restrictions. When the level of restrictions in the group with more restrictions is the highest (i.e., the multiplier is 100%), the loss in value added in the group with fewer restrictions without any lockdown is large: 18.6 trillion yen, or 3.51% of its pre-lockdown value added. These results clearly indicate that even when prefectures are not locked down, their economies can be damaged because of the propagation of the effect of the lockdowns in other prefectures through supply chains.

### 4.3 Effect of lifting the lockdown in one region

We further examine, how the recovery of a prefecture where lockdown is lifted is determined by its network characteristics, when only one prefecture lifts its lockdown and others remain locked down. We define the recovery rate of each prefecture as the ratio of the total gain of its value added or gross regional production (GRP) from lifting the lockdown to its total loss from the lockdown of all the prefectures for two weeks. The visualization of the recovery rate can be found in Fig 5 of [31]. See S6 Fig for the bar plot of the recovery rate of each prefecture.

One notable finding is that the prefectures that recover the most, including Hokkaido, Shimane, and Okinawa, which are remote from industrial hubs in terms of both geography and supply chains, suggesting the effect of network characteristics on economic recovery by lifting a lockdown. The name and location of each prefecture can be found in Fig A.2 of [31].

**Fig 4:**
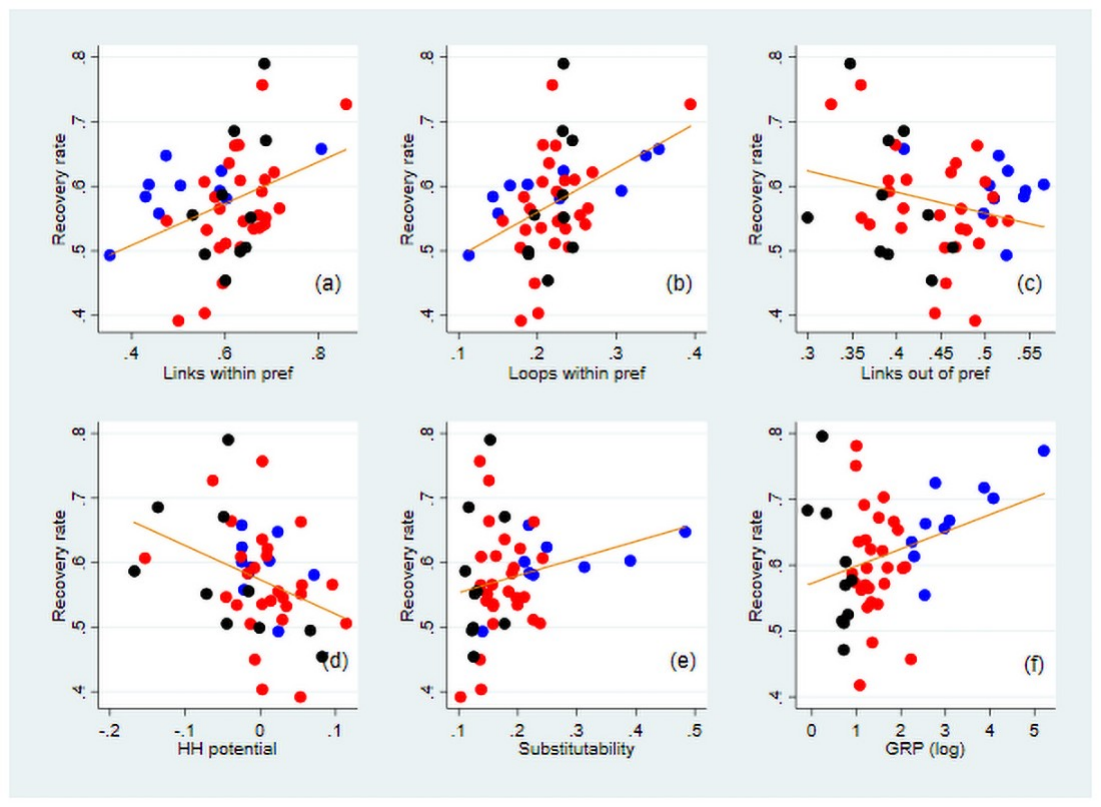
Correlation between the recovery rate and selected network measures. The vertical axis indicates the recovery rate, defined as the ratio of the increase in the GRP of a prefecture by lifting its own lockdown to its decrease because of the lockdown of all prefectures. Except for panel (f), the effect of GRP is excluded from the recovery rate. The horizontal axis indicates the share of the links within the prefecture to its all links in (a), the share of the loop flows within the prefecture to its total flows in (b), the share of the links to other prefectures to all links in (c), the standardised potential flows in (d), the share of substitutable suppliers to all suppliers outside the prefecture in (e), and GRP in logs in panel (f). The orange line in each panel specifies the fitted value from a linear regression that controls for the effect of GRP. The blue, black, and red dots show prefectures whose GRP is among the top 10, bottom 10, and others, respectively.

We further examine the correlation between the recovery rate and network measures explained in Section 3.3 (i.e. those for isolation, loops, upstreamness, and supplier substitution) and test the significance of the correlation using ordinary least squares (OLS) estimations. Fig 4 illustrates the correlation between the recovery rate and network measures. To control for the effect of the prefecture’s economic size on its recovery (Fig 4(f)), we include GRP in logs in all the OLS estimations and exclude the effect of GRP from the recovery rate in Fig 4. The number of links of each prefecture could also be controlled for; however, because its correlation coefficient with GRP is 0.965 (S3 Table), we do not use the total links in our regressions to avoid multicollinearity. S4 Table presents the OLS results.

In panels (a) and (b) of Fig 4, the supply-chain links and loops within the prefecture are found to be positively correlated with the recovery rate. These results suggest that when a prefecture is more isolated in the network and has more loops within it, the positive effect of lifting a lockdown circulates in the loops, which can mitigate the propagation of the negative effects of other prefectures’ lockdowns. By contrast, the outward links to other prefectures and the HH potential of the prefecture are negatively and significantly correlated with the recovery rate (panels (c) and (d)). These findings imply that prefectures with more upstream firms in supply chains tend to recover less from lifting their own lockdowns. Panel (e) indicates that the recovery rate is higher when more suppliers in other prefectures under lockdown can be replaced by those in the prefecture lifting its lockdown.

### 4.4 Effect of lifting the lockdowns in two regions simultaneously

**Fig 5:**
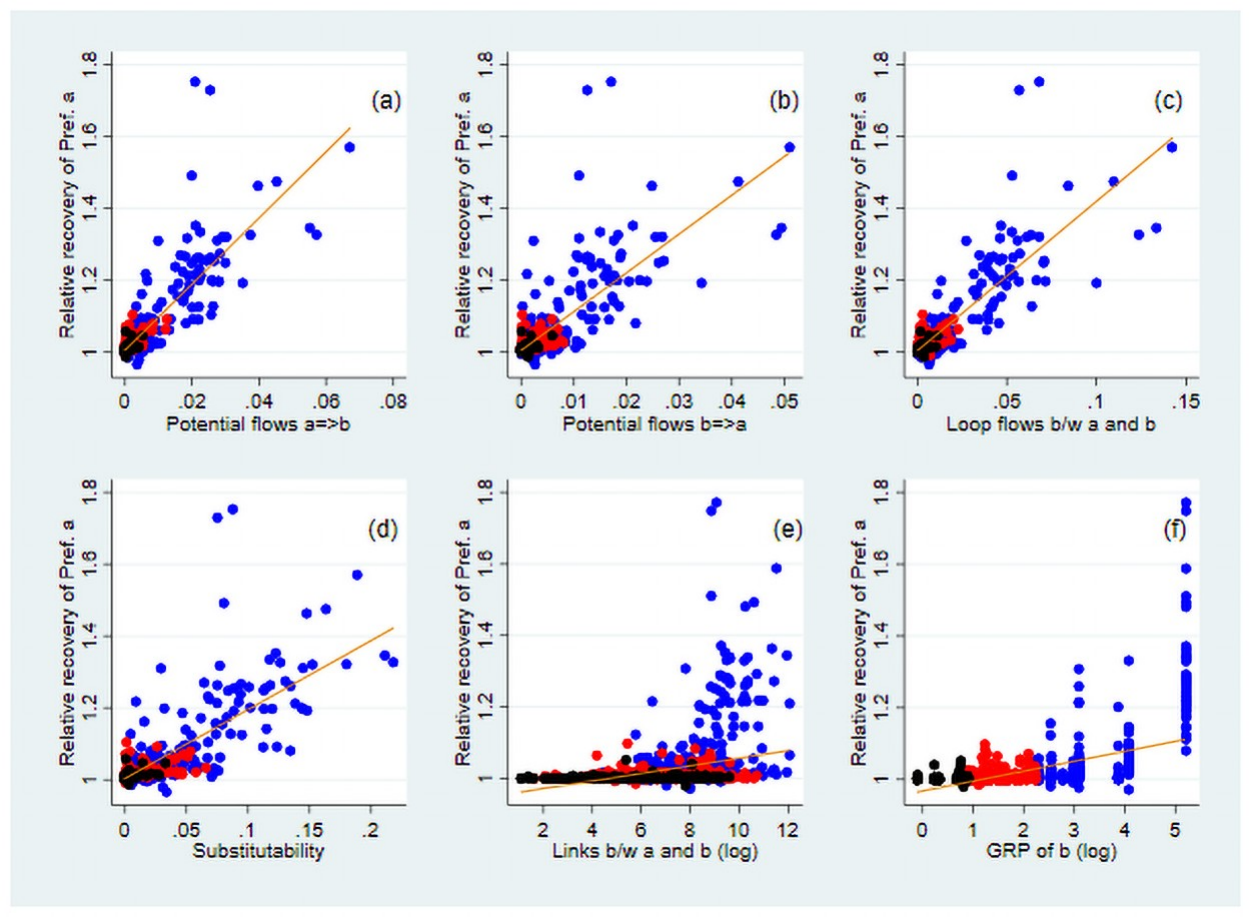
Correlation between the relative recovery and selected network measures. The vertical axis indicates the relative recovery of prefecture *a*, defined as the ratio of the increase in the GRP of prefecture *a* by lifting its lockdown together with prefecture *b* to its increase by lifting its lockdown alone. The effect of the GRP of *b* and total links between the two are excluded from the relative recovery measure. The variable in the horizontal axis is given by Eqs 3 and 4 in panels (a) and (b), respectively, Eq 5 in (c), the share of substitutable suppliers in *b* for those in *a* among *a*’s locked-down suppliers in (d), the number of links between prefectures *a* and *b* in (e) and the GRP of *b* in logs in (f). The orange line in each panel signifies the fitted value from a linear regression that controls for the effect of the GRP of *b* and total number of links between *a* and *b* in (a)–(d). The blue, black, and red dots show the pairs of prefectures *a* and *b* for which the GRP of *b* is among the top 10, bottom 10, and others, respectively.

Finally, we simulate the effect on the production of prefecture *a* if it lifted its lockdown together with prefecture *b*. We compare the recovery in prefecture *a*’s GRP by lifting its lockdown together with prefecture *b* and that by lifting its lockdown alone, and compute the relative recovery measure, as shown in S7 Fig. Using a regression framework as above, we investigate how the relative recovery measure of prefecture *a* is affected by the network relationships between prefectures *a* and *b*. Fig 5 illustrates the correlation between selected key variables and the relative recovery. In the regression analysis, we always control for the GRP of prefecture *b*, its squares, and the number of links between prefectures *a* and *b* that may affect the relative recovery (Fig 5(e) and 5(f)). Following this, we exclude these effects from the relative recovery in panels (a)–(d) in the figure. S6 Table presents the results of the OLS estimations.

Panels (a) and (b) of Fig 5 show that even after controlling for the effect of economic size and number of links between the two prefectures, the ratio of potential flows from prefecture *a* to *b* and from *b* to *a* to the total flows of *a* is positively correlated with the relative recovery. S8 Fig shows a similarly positive correlation for the number of links between the two, rather than potential flows, and the relative recovery. These results suggest that the recovery from lifting a lockdown is greater when two prefectures closely linked through their supply chains, regardless of the direction, lift their lockdowns together. Further, we find that prefecture *a* recovers more when prefectures *a* and *b* are linked through more circular flows (panel (c)), confirming that the positive impacts of lifting a lockdown can circulate and be strengthened in inter-regional supply-chain loops. Panel (d) indicates that if prefecture *a*’s suppliers in other prefectures are in lockdown but can be replaced by suppliers in prefecture *b* easily, prefecture *a*’s recovery is higher when the two prefectures lift their lockdowns together. Although the correlation between the relative recovery measure and network variables seems to be largely driven by the observations for which the GRP of prefecture *b* is large (depicted by the blue dots in Fig 5), we find that the positive correlation still exists without these observations (S9 Fig).

## 5 Discussion and conclusion

Our simulation analysis reveals that the economic effects of lockdowns in different regions interact with each other through supply chains. Our results and their implications can be summarised as follows.

First, when a firm is locked down, its suppliers and customer firms are affected because of a lack of demand and supply, respectively. Therefore, a region’s production can improve more if prefectures lift their lockdowns together when they are closely linked through supply chains in either direction (Fig 5(a) and 5(b)). In addition to the total number of links between the two regions, the intensity of such links compared with those with others is also important.

Second, when the firms in a region are in more upstream positions in the whole network or are predominantly suppliers of simple parts, the production of the region does not recover substantially by lifting its lockdown alone (Fig 4(d)). Although the negative economic effect of a lockdown can propagate downstream and upstream, firms can mitigate downstream propagation easily by using inventory or by replacing suppliers who are under lockdown. The difference between the downstream and upstream effects of lockdown is aggravated as the effect propagates further through supply chains. This finding is in line with the literature [36, 43] that also finds the upstream accumulation of negative effects on profits and assets. In practice, our result implies that a region with many small- and medium-sized suppliers of simple materials and parts should be cautious about whether it lifts its lockdown, which may not result in a large economic benefit but could still promote the spread of COVID-19.

Third, the production of a region can recover more by lifting its lockdown when it is more isolated in the network or embodies more supply-chain loops within the region (Fig 4(a) and 4(b)). Similarly, the production of the two regions can recover more by lifting their lockdowns together when their inter-regional links have more loops (Fig 5(c)). These results imply that the positive economic effect of lifting a lockdown circulates and is intensified in loops, consistent with those in [5]. Supply-chain loops exist between two regions when the final goods produced are used as inputs by suppliers, while suppliers provide parts and components to final-good producers and the loop stretches across two regions. The importance of loops in the diffusion of the economic effects in networks is not fully recognised, either in academic literature or in policymaking.

Finally, the recovery of a region from its lockdown is greater when suppliers who are still under lockdown can be replaced by those within the region or in other regions without a lockdown in place (Figs 4(e) and 5(f)). The role of the substitutability of suppliers in mitigating the propagation effect through supply chains has been empirically found in the literature [2, 5–7]. In practice, this finding suggests two management strategies for regional governments and firms. To minimise the economic loss from lockdown, a region should develop a full set of industries to allow for the possibility of the substitution of any industry. Alternatively, the firms in a region should be linked with geographically diverse suppliers so that suppliers in a region under lockdown can be replaced by those in other regions without a lockdown.

All these results point to the need for policy coordination among regions when regional governments impose or lift a lockdown. Although this study uses the inter-firm supply chains within a country and considers the economic effect of prefecture-level lockdowns, our results can be applied to examine the effect of country-level lockdowns propagating through international supply chains. For example, many suppliers of German firms are located in Eastern Europe and many suppliers of US firms are in Mexico. Our results thus suggest that the economic gains of Eastern Europe and Mexico from lifting their lockdowns are minimal if Germany and the United States, respectively, remain under lockdown. In addition, our framework can be applied to the case of other infectious diseases, and it is likely to suggest a need for the inter-regional and international coordination of lockdown strategies to prevent the spread of infection.

Since our model does not incorporate how lockdown strategies affect the spread of COVID-19, and because it is unclear how human and economic loss should be balanced to maximise social welfare, we cannot explicitly conclude in which cases a lockdown should be imposed or lifted. However, our analysis points to the importance of coordination between lockdown strategies among regions and countries that consider their economic effect in addition to their health effect.

## Supporting information

S1 Appendix Data. (PDF)

S2 Appendix Methods. (PDF)

S3 Appendix Results. (PDF)

S1 Fig An example of the HHD and loop and potential flow measures of prefectures. The left panel shows the supply chains of the six firms in the two prefectures. The right top and bottom panels present the potential flows and loop flows, respectively obtained from the HHD.
(PNG)

S2 Fig An example of the substitutability measure for two regions. The bottom shows the equation. *A*_*i*_ is the total number of suppliers outside prefectures *a* and *b*. The lowest two suppliers are applicable. A supplier in prefecture *b* belongs to the same industry as the upper firm of the outside suppliers, whereas the lower firm of the outside suppliers is not substitutable. Hence, *A*_*i*_ = 2 and *B*_*i*_ = 1.
(PNG)

S3 Fig Loss in value added as a percentage of total GDP, assuming different restriction levels for a lockdown of 14 days, between the groups with fewer and greater restrictions. A restriction level is defined by a multiplier for the sector-specific benchmark rates of reduction in production capacity. The red and blue parts of each bar show the loss of value added in the less and more restricted groups, respectively, as a percentage of GDP.
(PNG)

S4 Fig Loss in value added as a percentage of total GDP, assuming different restriction levels for a lockdown of 30 days, between the groups with fewer and greater restrictions. A restriction level is defined by a multiplier for the sector-specific benchmark rates of reduction in production capacity. The red and blue parts of each bar show the loss of value added in the less and more restricted groups, respectively as a percentage of GDP.
(PNG)

S5 Fig The ratio of the improvement in GDP by lifting the lockdown in each prefecture. The improvement is defined as the ratio of the increase in the national GDP by each prefecture lifting its lockdown to the decrease in GDP by all prefectures’ lockdowns. The horizontal axis indicates the JIS codes of the prefectures. The yellow, dark green, and light green bars show the ratio of the improvement when lockdowns persist for 14, 30, and 60 days, respectively.
(PNG)

S6 Fig Recovery rate in GRP by lifting the lockdown in each prefecture. The recovery rate is defined as the ratio of the increase in the GRP of each prefecture by lifting its lockdown to the decrease in its GRP by all prefectures’ lockdowns. The horizontal axis indicates the JIS codes of the prefectures. The yellow, dark green, and light green bars show the recovery rate when lockdowns persist for 14, 30, and 60 days, respectively.
(PNG)

S7 Fig Relative recovery from lifting the lockdown together to the recovery from lifting the lockdown alone. The relative recovery measure is defined as the ratio of the increase in the GRP of prefecture *a* when it lifts its lockdown together with prefecture *b* to its increase when prefecture *a* lifts its lockdown alone. The horizontal axis shows the JIS code of prefecture *a*. The colour of each dot indicates whether the GRP of prefecture *b* is among the top 10 (blue), the bottom 10 (black), or others (red).
(PNG)

S8 Fig Correlation between the relative recovery and selected network measures. The vertical axis indicates the relative recovery of prefecture *a*, defined as the ratio of the increase in the GRP of prefecture *a* by lifting its lockdown together with prefecture *b* to its increase by lifting its lockdown alone. The effect of the GRP of *b* and total links between the two are excluded from the relative recovery measure. The variable in the horizontal axis is given by Eqs 1 and 2 in panels (a) and (b), respectively. The orange line in each panel signifies the fitted value from a linear regression that controls for the effect of the GRP of *b* and total number of links between *a* and *b*. The blue, black, and red dots indicate the pairs of prefectures *a* and *b* for which the GRP of *b* is among the top 10, bottom 10, and others, respectively.
(PNG)

S9 Fig Correlation between the relative recovery and selected network measures. See the caption of Fig 5 and S8 Fig. for the definitions of the variables used here. The green line in each panel signifies the fitted value from a linear regression that controls for the effect of the GRP of *b* and total number of links between *a* and *b* in (a)–(g). The black and red dots indicate the pairs of prefectures *a* and *b* for which the GRP of *b* is among the bottom 10 and between 11 and 37, respectively.
(PNG)

S10 Fig Correlation between the recovery rate and selected network measures. See the caption of Fig 4 for the definitions of the variables used here. The orange line in each panel specifies the fitted value from a linear regression that controls for the effect of GRP in (b)–(f). The blue, black, and red dots indicate the prefectures whose GRP is among the top 10, the bottom 10, or others, respectively.
(PNG)

S11 Fig Correlation between the relative recovery and selected network measures. See the caption of Fig 5 for the definitions of the variables used here. The red line in each panel signifies the fitted value from a linear regression that controls for the effect of the GRP of *b* and total number of links between *a* and *b* in (a)–(g). The blue, black, and red dots indicate the pairs of prefectures *a* and *b* for which the GRP of *b* is among the top 10, the bottom 10, or others, respectively.
(PNG)

S1 Table Sector-specific rates of reduction in production capacity. Sectors are classified by the JSIC [23] at the two-digit level, except for industries 560, 561, and 569 for which we use three-digit codes to reflect the actual circumstances. The sector names are abbreviated. S1 Table lists the sector descriptions and abbreviations.
(PDF)

S2 Table Sector classifications and abbreviations. (PDF)

S3 Table Correlation matrix of the variables used in Section 4.3. The definitions of the variables are as follows. RecRatio: the recovery rate defined as the ratio of the increase in the GRP of each prefecture by lifting its lockdown to the decrease in its GRP by all prefectures’ lockdowns. GRP: gross regional product (log). Links: the degree (log). InLink: the share of links within the prefecture to all its links. InLoop: the share of loop flows within the prefecture to all its flows. OutLink: the share of outward inter-prefectural links to all the links of the prefecture. Potential: the average HH potential of the firms in the prefecture. Sub: the share of substitutable suppliers to all suppliers of the prefecture located outside the prefecture.
(PDF)

S4 Table Regression results for Section 4.3. The dependent variable is the recovery rate. See the caption of Table S3 Table for the definitions of the independent variables. Standard errors are in parentheses. *** p<0.01, ** p<0.05, * p<0.1.
(PDF)

S5 Table Correlation matrix of the variables used in Section 4.4. The definitions of the variables are as follows. *Recov*_*a*_: the relative recovery of prefecture *a* defined as the ratio of the increase in the GRP of prefecture *a* by lifting its lockdown together with prefecture *b* to its increase by lifting its lockdown alone. *Link*_*ab*_: the share of links from *a* to *b* to all links from *a*. *Link*_*ba*_: the share of links from *b* to *a* to all links from *a*. *Pot*_*ab*_: the share of potential flows from *b* to *a* to the total links of *a*. *Pot*_*ba*_: the share of potential flows from *a* to *b* to the total links of *a*. *Sub*_*ab*_: the share of suppliers substitutable by those in *b* to *a*’s suppliers outside *a* and *b*. *Sub*_*ba*_: the share of suppliers substitutable by those in *a* to *b*’s suppliers outside *a* and *b*. *Loop*_*ab*_: the share of loop flows between *a* and *b* to the total flows between the two. *Bi*_*ab*_: the number of inter-prefecture links between *a* and *b* in logs. *GRP*_*j*_: GRP of *b* in logs.
(PDF)

S6 Table Regression results for Section 4.4. The dependent variable is the relative recovery measure. See the caption of Table S5 Table for the definitions of the independent variables. Standard errors are in parentheses. *** p<0.01, ** p<0.05, * p<0.1.
(PDF)
