# Supplementary material for: Do economic effects of the anti-COVID-19 lockdowns in different regions interact through supply chains?
Source: PLoS One. 2021 Jul 30;16(7):e0255031. doi: 10.1371/journal.pone.0255031 (PMC8323942; doi:10.1371/journal.pone.0255031)
Supplement: S1 Appendix — (PDF) [file pone.0255031.s001.pdf]

S1 Appendix. Data

Supply chains In the TSR data, the maximum number of suppliers and customers reported by each firm is 24. However, we can capture more than 24 by looking at the supplier–customer relations from the opposite direction. Because the TSR data include the addresses of the headquarters of each firm, we can identify the longitude and latitude of each headquarter using the geocoding service provided by the Center for Spatial Information Science at the University of Tokyo.

Because the TSR data do not include the value of each transaction between two firms, we estimate it in two steps. First, we divide each supplier's sales into its customers in proportion to the sales of customers to obtain a tentative sales value. Second, we employ the 2015 IO Tables for Japan [1] to transform these tentative values into more realistic ones. Specifically, we aggregate the tentative values at the firm-pair level to obtain the total sales for each pair of sectors. We then divide the total sales for each sector pair by the transaction values for the corresponding pair in the IO tables. The ratio is then used to estimate the transaction values between firms. The final consumption of each sector is allocated to all the firms in the sector using their sales as weights.

Although the supply chains used in our simulations are at the firm level, this study often uses features of the supply chains at the prefecture level because different prefectures imposed lockdowns to different degrees. We observe that Tokyo is the centre of supply chains in Japan. The visualization on the map be found in Figure A.1 of [2]. In addition, several smaller hubs such as Aichi, Osaka, and Fukuoka also exist.

Prefectures in Japan As this study uses prefectures as the unit of regions, it is important to provide information on prefectures in Japan. The locations, names, and JIS codes can be found in Figure A.2 of [2]. In S5 and S6 Figs, the JIS codes are shown on the horizontal axis.

References

1. Ministry of Internal Affairs and Communications, the Cabinet Office, the Financial Services Agency, the Ministry of Finance, the Ministry of Education, Culture, Sports, Science and Technology, the Ministry of Health, Labour and Welfare, the Ministry of Agriculture, Forestry and Fisheries, the Ministry of Economy, Trade and Industry, the Ministry of Land, Infrastructure, Transport and Tourism, and the Ministry of Environment, Japan. 2015 Input-Output tables for Japan. `https://www.soumu.go.jp/english/dgpp_ss/data/io/index.htm`, 2015.

2. Hiroyasu Inoue, Yohsuke Murase, and Todo Yasuyuki. Do economic effects of the "anti-covid-19" lockdowns in different regions interact through supply chains? SSRN 3692937, Social Science Research Network, 2021.
