# Supplementary material for: Do economic effects of the anti-COVID-19 lockdowns in different regions interact through supply chains?
Source: PLoS One. 2021 Jul 30;16(7):e0255031. doi: 10.1371/journal.pone.0255031 (PMC8323942; doi:10.1371/journal.pone.0255031)
Supplement: S2 Appendix — (PDF) [file pone.0255031.s002.pdf]

S2 Appendix. Methods

Model We rely on the model of Inoue and Todo [1,2], an extension of the existing agent-based models used to examine the propagation of shocks by natural disasters through supply chains, including Hallegatte's model [3]. Each firm uses a variety of intermediates as inputs and delivers a sector-specific product to other firms and final consumers. Firms have an inventory of intermediates to address possible supply shortages.

In the initial stage before an economic shock, the daily trade volume from supplier j to customer i is denoted by $A_{i,j}$, whereas the daily trade volume from firm i to final consumers is denoted by C_i. Then, the initial production of firm i in a day is given by

$$P_{\text{ini}i} = \Sigma_j A_{j,i} + C_i. \tag{1}$$

On day t after the initial stage, the previous day's demand for firm i's product is $D_i^*(t-1)$. The firm thus makes orders to each supplier j so that the amount of its product from the supplier j can meet this demand, $A_{i,j} D_i^*(t-1)/P_{\text{ini}i}$. We assume that firm i has an inventory of the intermediate goods produced by firm j on day t, $S_{i,j}(t)$, and aims to restore this inventory to a level equal to a given number of days n_i of the utilisation of the product of supplier j. The constant n_i is assumed to be Poisson distributed, where its mean is n, which is a parameter. In addition, n_i does not take a number smaller than 4, although the model in the previous literature sets this number to 2. Since the small minimum inventory size causes a bullwhip effect (fluctuation of production level), we set the number to 4 in this study and recalibrate the parameters. When the actual inventory is smaller than its target, firm i increases its inventory gradually by $1/\tau$ of the gap, so that it reaches the target in τ days, where τ is assumed to be 6 to follow the original model [3]. Therefore, the order from firm i to its supplier j on day t, denoted by $O_{i,j}(t)$, is given by

$$O_{i,j}(t) = A_{i,j} \frac{D_i^*(t-1)}{P_{\text{ini}i}} + \frac{1}{\tau} \left[n_i A_{i,j} - S_{i,j}(t) \right], \tag{2}$$

where the inventory gap is in brackets. Accordingly, total demand for the product of supplier i on day t, $D_i(t)$, is given by the sum of final demand from the final consumers and the total orders from customers:

$$D_i(t) = \Sigma_j O_{j,i}(t) + C_i. \tag{3}$$

Now, suppose that an economic shock hits the economy on day 0, and that firm i is directly affected. Subsequently, the proportion $\delta_i(t)$ of the production capital of firm i is malfunctioning. In this study, δ_i is determined by the sector and prefecture to which firm i belongs, and the duration for which a lockdown is imposed. Hence, the production capacity of firm i, defined as its maximum production assuming no supply shortages, $P_{\text{cap}i}(t)$, is given by

$$P_{\text{cap}i}(t) = P_{\text{ini}i}(1 - \delta_i(t)). \tag{4}$$

The production of firm i might also be limited by the shortage of supplies. Because we assume that firms in the same sector produce the same product, the shortage of supplies suffered by firm j in sector s can be compensated for by supplies from firm k in the same sector s. Firms cannot substitute new suppliers for affected suppliers after the disaster, as we assume fixed supply chains. Thus, the total inventory of the products delivered by firms in sector s in firm i on day t is

$$S_{\text{tot}i,s}(t) = \Sigma_{j \in s} S_{i,j}(t). \tag{5}$$

The initial consumption of products in sector s of firm i before the disaster is also defined for convenience:

$$A_{\text{tot}i,s} = \Sigma_{j \in s} A_{i,j}. \tag{6}$$

The maximum possible production of firm i limited by the inventory of product of sector s on day t, $P_{\text{pro}i,s}(t)$, is given by

$$P_{\text{pro}i,s}(t) = \frac{S_{\text{tot}i,s}(t)}{A_{\text{tot}i,s}} P_{\text{ini}i}. \tag{7}$$

Then, we can determine the maximum production of firm i on day t, considering its production capacity, $P_{\text{cap}i}(t)$, and its production constraints due to the shortage of supplies, $P_{\text{pro}i,s}(t)$:

$$P_{\text{max}i}(t) = \text{Min}\left(P_{\text{cap}i}(t), \text{Min}_s(P_{\text{pro}i,s}(t))\right). \tag{8}$$

Therefore, the actual production of firm i on day t is given by

$$P_{\text{act}i}(t) = \text{Min}\left(P_{\text{max}i}(t), D_i(t)\right). \tag{9}$$

When the demand for a firm is greater than its production capacity, the firm cannot completely satisfy its demand, as denoted by Equation (4). In this case, firms should ration their product to their customers. We propose a rationing policy in which customers and final consumers are prioritised if they have orders that are smaller than their initial orders, instead of being treated equally, as in the previous work [3].

Suppose that firm i has customers j and a final consumer. Then, the ratios of the order from customers j and the final consumer after the shock to the one before the shock denoted by $O^{rel}_{j,i}$ and O^{rel}_{c}, respectively are determined by the following steps, where $O^{sub}_{j,i}$ and O^{sub}_{c} are temporal variables used to calculate the realised order and are set to be zero initially.

1. Obtain the remaining production r of firm i
2. Calculate $O^{rel}_{\min} = \mathrm{Min}(O^{rel}_{j,i}, O^{rel}_{c})$
3. If $r \leq (\sum_j O^{rel}_{\min} O_{j,i} + O^{rel}_{\min} C_i)$ then proceed to 8
4. Add $O^{rel}_{\min}$ to $O^{sub}_{j,i}$ and O^{sub}_{c}
5. Subtract $(\sum_j O^{rel}_{\min} O_{j,i} + O^{rel}_{\min} C_i)$ from r
6. Remove the customer or the final consumer that indicated $O^{rel}_{\min}$ from the calculation
7. Return to Step 2
8. Calculate O^{rea} that satisfies $r = (\sum_j O^{rea} O_{j,i} + O^{rea} C_i)$
9. Obtain $O^{*}_{j,i} = O^{rea} O_{j,i} + O^{sub}_{j,i} O_{j,i}$ and $C^{*}_{i} = O^{rea} C_i + O^{sub}_{c} C_i$, where the realised order from firm j to supplier i is denoted by $O^{*}_{j,i}(t)$, and the realised order from a final consumer is C^{*}_{i}
10. Finalise the calculation

Under this rationing policy, total realised demand for firm i, $D^{*}_{i}(t)$, is given by

$$D^{*}_{i}(t) = \Sigma_j O^{*}_{i,j}(t) + C^{*}_{i}, \tag{10}$$

where the realised order from firm i to supplier j is denoted by $O^{*}_{i,j}(t)$ and that from the final consumers is C^{*}_{i}. According to firms' production and procurement activities on day t, the inventory of firm j's product in firm i on day $t+1$ is updated to

$$S_{i,j}(t+1) = S_{i,j}(t) + O^{*}_{i,j}(t) - A_{i,j} \frac{P_{\mathrm{act}i}(t-1)}{P_{\mathrm{ini}i}}. \tag{11}$$

Several caveats of this model and data should be mentioned. First, we assume that firms cannot find a new supplier when facing a shortage from their current suppliers. Second, for simplicity, our model assumes that inputs from the service sector can be stored as inventory, just like inputs from manufacturing. Third, our model ignores changes in the prices of products and wages of labour incorporated in [4,5] and focuses on the dynamics of production because of supply-chain disruptions. Fourth, the TSR data report only the location of the headquarters of each firm, and not the location of its branches. Because the headquarters of firms are concentrated in Tokyo, production activities in Tokyo are most likely to be overvalued in our analysis. Fifth, because of data limitations, we ignore the international supply-chain links in our simulations. Finally, this study ignores the impacts of COVID-19 on human and firm behaviours in the post-COVID period. These behavioural changes may influence consumption and production that are assumed to remain the same in this period.

Sectoral differences in production capacity after lockdowns No data for production capacity (i.e., Pcap in the model) during the lockdown in Japan at the firm or sector level are available. Although the Indices of All Industry Activities (IAIA) provides data for *post-lockdown production* at the sector level (Section 3.3), or Pact in our model as averaged within a sector, we require information about *production capacity*, Pcap. Therefore, we assume that the rate of reduction in production capacity for each sector is given by the degree of the reduction from exposure to the virus [6] multiplied by the share of workers who cannot work from home [7] (Section 3.3). The rate of reduction from exposure to the virus is determined by how the workers in the sector have to reduce their activities to avoid contact with others to prevent infection. As [9] defines the rate of reduction uniformly worldwide, we modify the rate for some sectors that clearly differ from the practice in Japan. S1 Table. shows the rates of reduction for each sector assumed in our simulations.

Helmholtz-Hodge decomposition The Helmholtz-Hodge decomposition (HHD) decomposes a flow from a node to another in a network into a potential flow component and a loop flow component. A potential flow component is determined by the upstream/downstream location of the node in a network [8], whereas a loop flow component is given by a constraint such that the summation of the incoming and outgoing loop flows of all the nodes equals zero. This method has been used to find the structure of potential and loop flows in complex networks. See, for example, [9-12].

Suppose we have a flow of a matrix denoted by B_{ij} such that a flow from node i to node j is represented by B_{ij}. For simplicity, we assume $\forall i, j\ B_{ij} \geq 0$. A_{ij} is a binary adjacency matrix generated from B_{ij}:

$$A_{ij} = \begin{array}{ll} 1 & \text{if } B_{ij} > 0, \\ 0 & \text{otherwise.} \end{array} \tag{12}$$

We define a 'net flow' F_{ij} by

$$F_{ij} = B_{ij} - B_{ji}, \tag{13}$$

and a 'net weight' w_{ij} by

$$w_{ij} = A_{ij} + A_{ji}. \tag{14}$$

Note that w_{ij} is symmetric, $w_{ij} = w_{ji}$, and non-negative, $w_{ij} \geq 0$, for any pair of i and j.

Then, the HHD is given by

$$F_{ij} = F_{ij}^{(c)} + F_{ij}^{(p)}, \tag{15}$$

where the loop flow $F_{ij}^{(c)}$ satisfies

$$\sum_j F_{ij}^{(c)} = 0, \tag{16}$$

meaning that loop flows are divergence-free. The potential flow, $F_{ij}^{(p)}$, can be expressed as

$$F_{ij}^{(p)} = w_{ij}(\phi_i - \phi_j), \tag{17}$$

where ϕ_i is the Helmholtz-Hodge (HH) potential of node i that identifies its upstream/downstream position in the network. More precisely, ϕ_i is larger when node i is located in a more upstream position in the network and vice versa. Equation (17)

indicates that the potential flow $F_{ij}^{(p)}$ is the difference in the HH potential between two nodes when the two are linked and zero when they are not linked. We further assume

$$\sum_i \phi_i = 0 \tag{18}$$

for normalisation purposes. Then, equations (15)–(18) can be uniquely solved for $F_{ij}^{(c)}$, $F_{ij}^{(p)}$, and ϕ_i for all i and j in the whole network.

S1 Fig. shows a simple example to explain the intuition behind the potential and loop flows, where potential is obtained from the HHD, and potential and loop flow measures between two prefectures (i.e., Pot_{ab}, Pot_{ba}, and $Loop_{ab}$ are defined in Section 4.4). The left panel shows a supply chain with six firms in prefectures a and b. The right top and bottom panels indicate the potential flows and loop flows, respectively decomposed by the HHD. The numbers in red in the right top panel represent the HH potential, or the upstreamness in supply chains, for each firm. Although there is no 'loop' in a standard sense among the firms in this example, the HHD identifies loop flows in the sense that the nodes in the loop are affected by each other. Hence, shocks circulate in the loop and work differently from those in the non-loop potential flows.

Specifically, Pot_{ab} is the sum of the total potential flows from the firms in prefecture a to those in prefecture b (there is only a potential flow from prefectures a to b in this example), divided by the total number of flows of firms in prefecture a. Therefore, $Pot_{ab} = (2/3)/4 = 1/6$. Pot_{ba} is the opposite direction and $Pot_{ba} = 1/6$. $Loop_{ab}$ is the sum of the total loop flows between the firms in prefectures a and b. Thus, there are two loop flows between a and b in this example, $Loop_{ab} = (2/3)/4 = 1/6$ and, similarly, $Loop_{ba} = 1/6$.

The average of the HH potential ϕ_i of the firms in the supply-chain network, which is normalised so that its overall average is zero, is calculated for each prefecture. We see the large variation in the upstreamness of the firms at the prefecture level. The visualization on the map can be found in Figure B.2 of [13].

Substitutability for two regions Since the definition of the substitutability measure for two regions is not as simple as the definition for one region, we provide a further explanation. S2 Fig. is an example for the suppliers of a firm in prefecture a. The substitutability of prefecture a by prefecture b is a fraction. The denominator is the total number of suppliers that deliver goods to the firms in prefecture a except suppliers in prefecture a or b. (We call this A_i in the figure.) Hereafter, a supplier implies a supplier of a firm in prefecture a. The numerator is the total number of substitutable suppliers in A_i. A supplier in A_i is substitutable if a supplier in prefecture b belongs to the same industry as the focal supplier.

References

1. Hiroyasu Inoue and Yasuyuki Todo. Firm-level propagation of shocks through supply-chain networks. *Nature Sustainability*, 2:841–847, 2019.

2. Hiroyasu Inoue and Yasuyuki Todo. Propagation of negative shocks through firm networks: Evidence from simulation on comprehensive supply-chain data. *PLoS ONE*, 14(3), 2019.

3. Stéphane Hallegatte. An adaptive regional input-output model and its application to the assessment of the economic cost of Katrina. *Risk analysis*, 28(3):779–799, 2008.

4 C. Otto, S.N. Willner, L. Wenz, K. Frieler, and A. Levermann. Modeling loss-propagation in the global supply network: The dynamic agent-based model acclimate. *Journal of Economic Dynamics and Control*, 83:232–269, 2017.

5. Celian Colon, Stephane Hallegatte, and Julie Rozenberg. *Transportation and Supply Chain Resilience in the United Republic of Tanzania: Assessing the Supply-Chain Impacts of Disaster-Induced Transportation Disruptions*. World Bank, 2019.

6. Barthélémy Bonadio, Zhen Huo, Andrei A Levchenko, and Nitya Pandalai-Nayar. Global supply chains in the pandemic. NBER Working Paper Series No. 27224, National Bureau of Economic Research, 2020.

7. Dabo Guan, Daoping Wang, Stephane Hallegatte, Jingwen Huo, Shuping Li, Yangchun Bai, Tianyang Lei, Qianyu Xue, Steven J Davis, D'Maris Coffman, et al. Global economic footprint of the covid-19 pandemic. *Nature Human Behavior*, 4:577–587, 2020.

8. X. Jiang, L.-H. Lim, Y. Yao, and Y. Ye. Statistical ranking and combinatorial hodge theory. *Mathematical Programming*, 127(1):203–244, 2011.

9. Yuichi Kichikawa, Hiroshi Iyetomi, Takashi Iino, and Hiroyasu Inoue. Community structure based on circular flow in a large-scale transaction network. *Applied Network Science*, 4(1):92, 2019.

10. H. Iyetomi, H. Aoyama, Y. Fujiwara, W. Souma, I. Vodenska, and H. Yoshikawa. Relationship between macroeconomic indicators and economic cycles in u.s. *Sci. Rep.*, 10:8420, 2020. https://doi.org/10.1038/s41598-020-65002-3.

11. RS MacKay, S Johnson, and B Sansom. How directed is a directed network? *arXiv preprint arXiv:2001.05173*, 2020.

12. Y. Fujiwara, H. Inoue, T. Yamaguchi, H. Aoyama, and T. Tanaka. Money flow network among firms' accounts in a regional bank of japan. `https://ssrn.com/abstract=3662893`, July 2020.

13. Hiroyasu Inoue, Yohsuke Murase, and Todo Yasuyuki. Do economic effects of the "anti-covid-19" lockdowns in different regions interact through supply chains? SSRN 3692937, Social Science Research Network, 2021.
