# Supplementary material for: Do economic effects of the anti-COVID-19 lockdowns in different regions interact through supply chains?
Source: PLoS One. 2021 Jul 30;16(7):e0255031. doi: 10.1371/journal.pone.0255031 (PMC8323942; doi:10.1371/journal.pone.0255031)
Supplement: S3 Appendix — (PDF) [file pone.0255031.s003.pdf]

S3 Appendix. Results

Simulation of the effect of the actual lockdown A video is available for the temporal and geographical visualisation of the lockdown simulation at `https://youtu.be/q029a_e1akU`. The map in the video indicates the rate of reduction in firm production averaged within each municipality. The red areas indicate that the production in the area is less than or equal to 20% of firms' capacity on average, whereas the light red and orange areas show firms with a more moderate decline in production. The inset in the video indicates Figure 2 and the number of days from the first lockdown. The visualisation clearly shows the areas that are not under lockdown are also affected by lockdowns in other areas. For example, from day 0 to day 8, only seven prefectures are under lockdown but most areas in Japan are affected (see Section 3.2). This reduction in production occurs because the demand reduction propagates to the suppliers without any buffer. However, supply reduction can be mitigated because each client holds inventories for the intermediate goods.

Estimation of daily GDP from IAIA The IAIA indicates the changes in production in all industries in Japan, compared with those in the previous month and in the same month in the previous year, based on firm surveys [1]. We assume that the daily production on 7 April (day 0) is the same as that in March and thus can be calculated from the IAIA in March. Then, we estimate the daily GDP in April (or May) by (yearly GDP)/365$\times$(IAIA in April (May))/(IAIA in March) and illustrate it in the left (right) red line in Figure 2.

Interconnected effect of the different strictness of regional lockdowns In Section 4.2, we show that the different levels of lockdown strictness between the groups with fewer or greater restrictions affect the economic losses of both the two groups, particularly assuming that the lockdown continues for 60 days. We also experiment with different lockdown durations (14 and 30 days) and present the results in S3 and S4 Figs. The main result that the strictness of the lockdown in the group with greater restrictions that includes the major industrial clusters substantially affects the economic loss of the other group by propagation through supply chains, still holds.

Effect of lifting the lockdown in one region Section 4.3 presents the effect of lifting the lockdown in a prefecture on its production, assuming that all the other prefectures are still under lockdown. S5 Fig. shows the ratio of the increase in national GDP from each prefecture lifting its lockdown to the decrease in GDP by all prefectures' lockdowns. The prefectures are horizontally aligned in order of JIS cods. The top three prefectures in terms of recovery rate are Tokyo, Osaka, and Fukuoka.

S6 Fig. illustrates the ratio of increase in the value added production, or gross regional product (GRP), of each prefecture by lifting its lockdown to the decrease in its GRP by all prefectures' lockdowns.

Regression analyses In Section 4.3, we conducted regression analyses to examine what attributes of prefectures cause a larger economic recovery by lifting the lockdown in only one prefecture, using Ordinary Least Squares (OLS) models. S3 Table. shows the correlation coefficients between all the variables used in the regression analysis and S4 Table. presents the detailed regression results.

In Section 4.4, we conducted regression analyses to examine what attributes of prefectures cause a larger economic recovery by lifting the lockdown in two prefectures simultaneously, using OLS models. The relative recovery measure defined as the ratio of the increase in the GRP of prefecture a when it lifts its lockdown together with prefecture b to its increase when prefecture a lifts its lockdown alone. S5 Table. shows the correlation coefficients between all the variables used in the regression analysis and S6 Table. presents the detailed regression results.

To check the robustness of our main results, we experimented with different rates of reduction in production capacity, where we assume the share of working from home is zero for all the sectors in S3. Appendix. In other words, in this alternative simulation analysis, we assume a stricter level of lockdown. S10 and S11 Figs. present the results, which are essentially the same as our benchmark results in Figures 4 and 5.

Reference

1. Ministry of Economy, Trade, and Industry. Indices of all industry production. `https://www.meti.go.jp/english/statistics/tyo/zenkatu/index.html`, 2020.
