# Supplementary figures and images for: Do economic effects of the anti-COVID-19 lockdowns in different regions interact through supply chains?
Source: PLoS One. 2021 Jul 30;16(7):e0255031. doi: 10.1371/journal.pone.0255031 (PMC8323942; doi:10.1371/journal.pone.0255031)

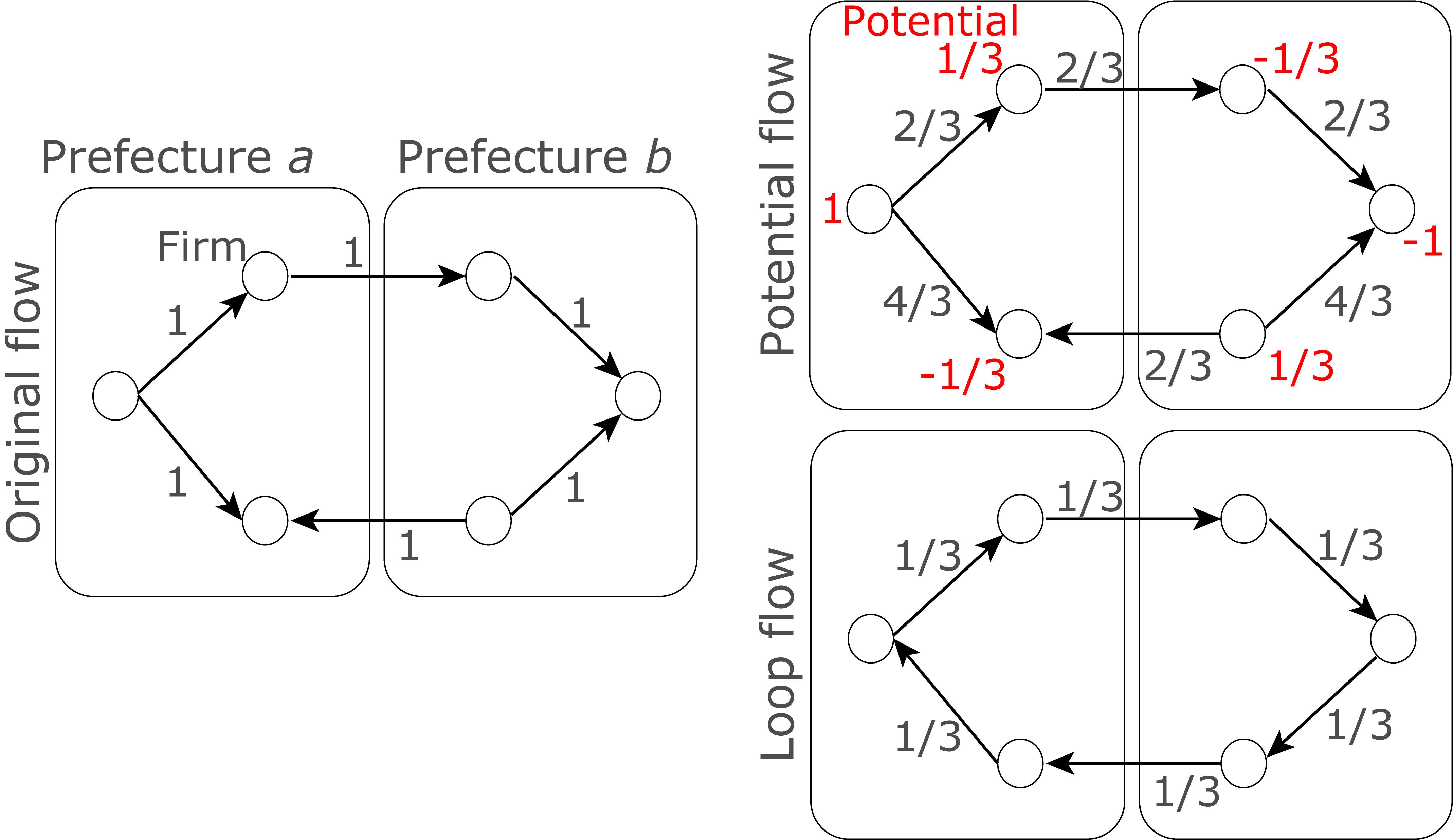

Supplement: S1 Fig — The left panel shows the supply chains of the six firms in the two prefectures. The right top and bottom panels present the potential flows and loop flows, respectively obtained from the HHD. (PNG) [file pone.0255031.s004.png]

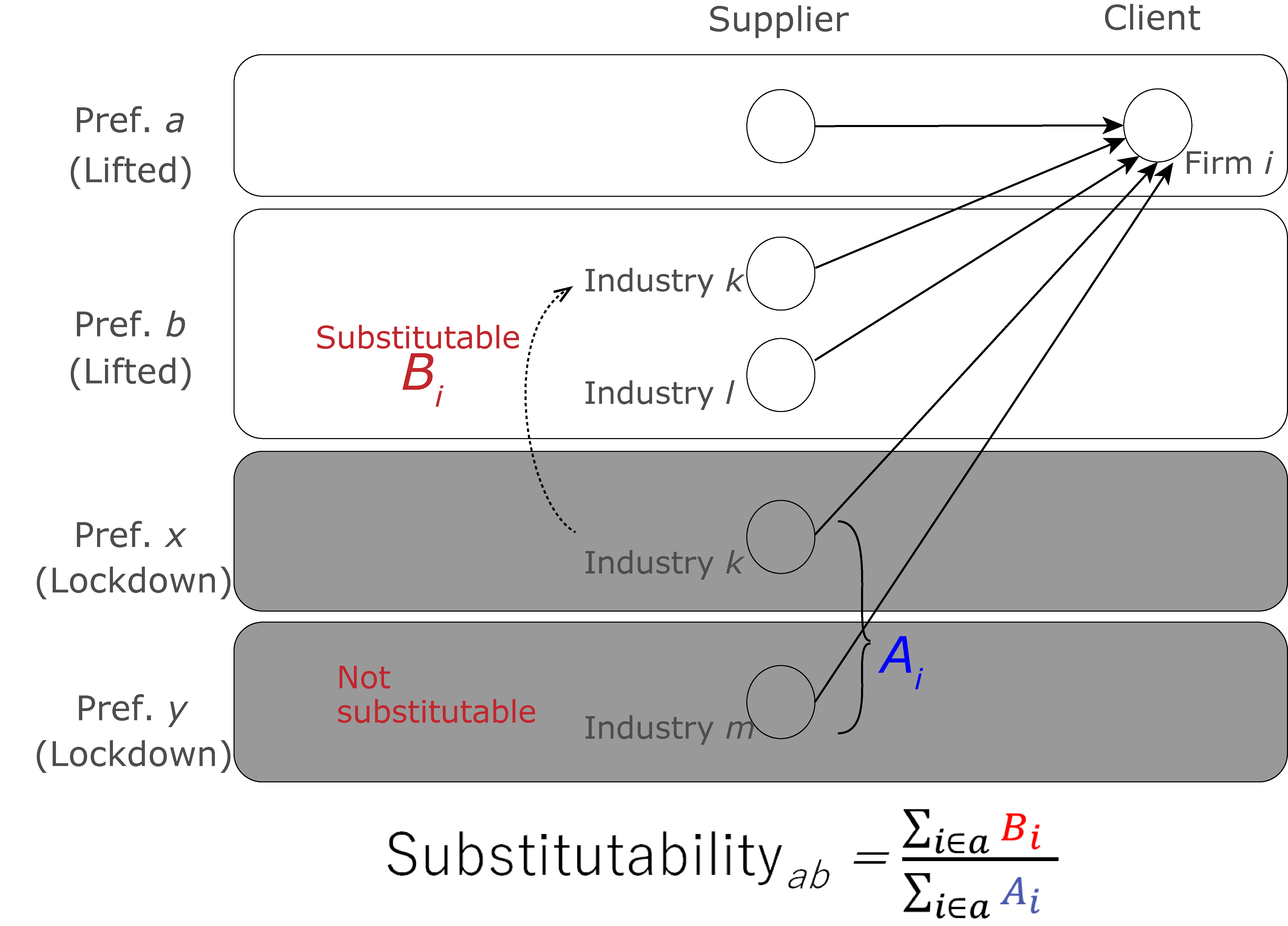

Supplement: S2 Fig — The bottom shows the equation. Ai is the total number of suppliers outside prefectures a and b. The lowest two suppliers are applicable. A supplier in prefecture b belongs to the same industry as the upper firm of the outside suppliers, whereas the lower firm of the outside suppliers is not substitutable. Hence, Ai = 2 and Bi = 1. (PNG) [file pone.0255031.s005.png]

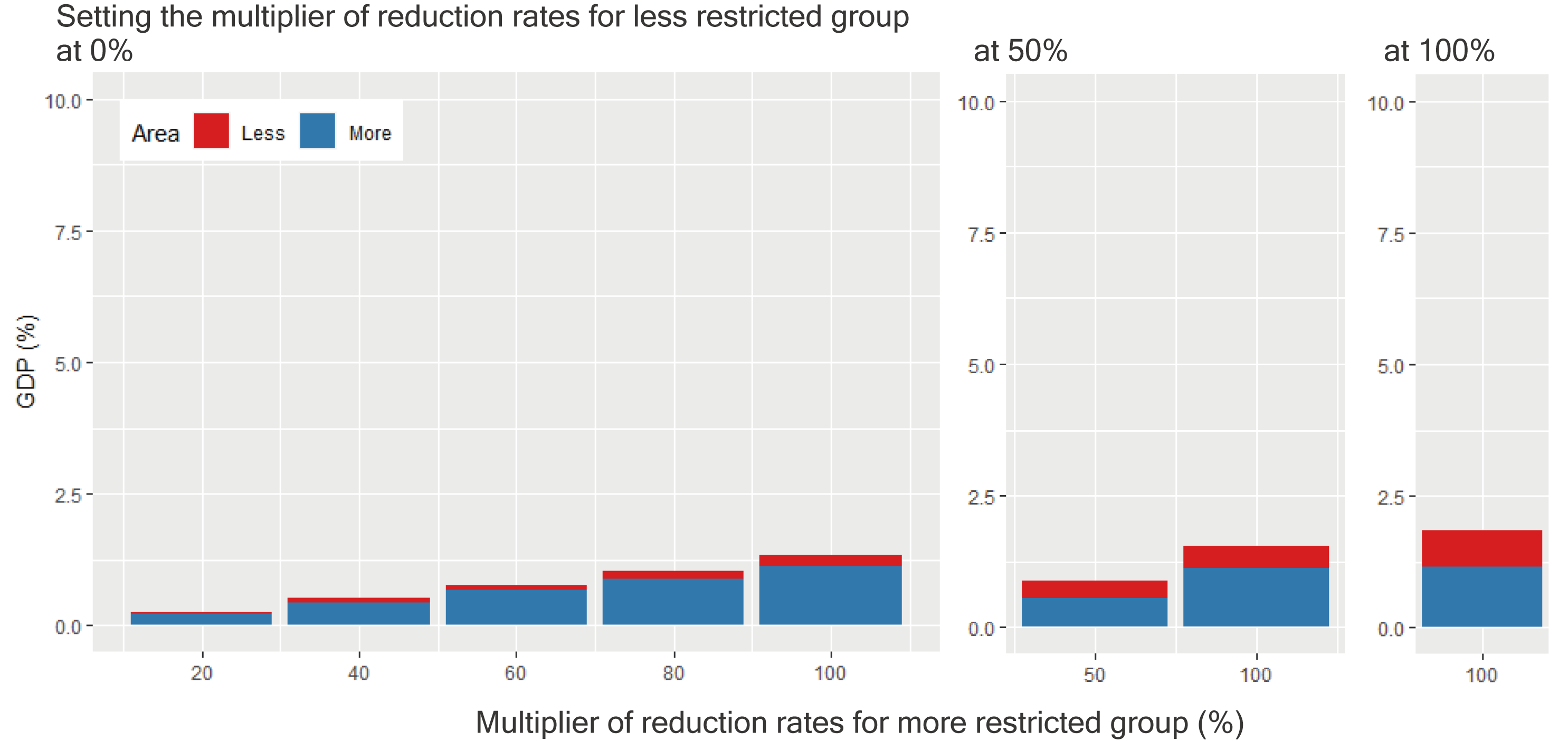

Supplement: S3 Fig — A restriction level is defined by a multiplier for the sector-specific benchmark rates of reduction in production capacity. The red and blue parts of each bar show the loss of value added in the less and more restricted groups, respectively, as a percentage of GDP. (PNG) [file pone.0255031.s006.png]

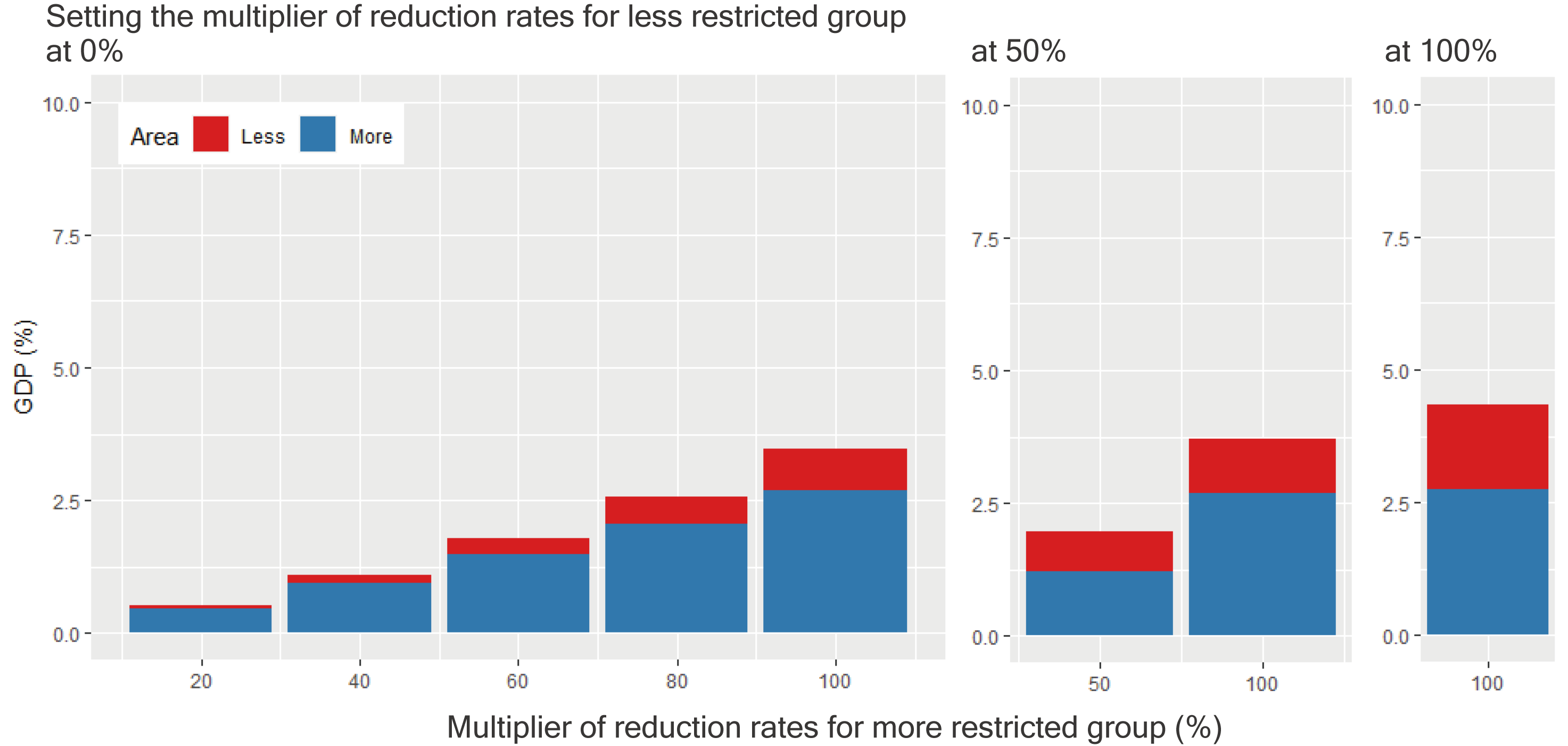

Supplement: S4 Fig — A restriction level is defined by a multiplier for the sector-specific benchmark rates of reduction in production capacity. The red and blue parts of each bar show the loss of value added in the less and more restricted groups, respectively as a percentage of GDP. (PNG) [file pone.0255031.s007.png]

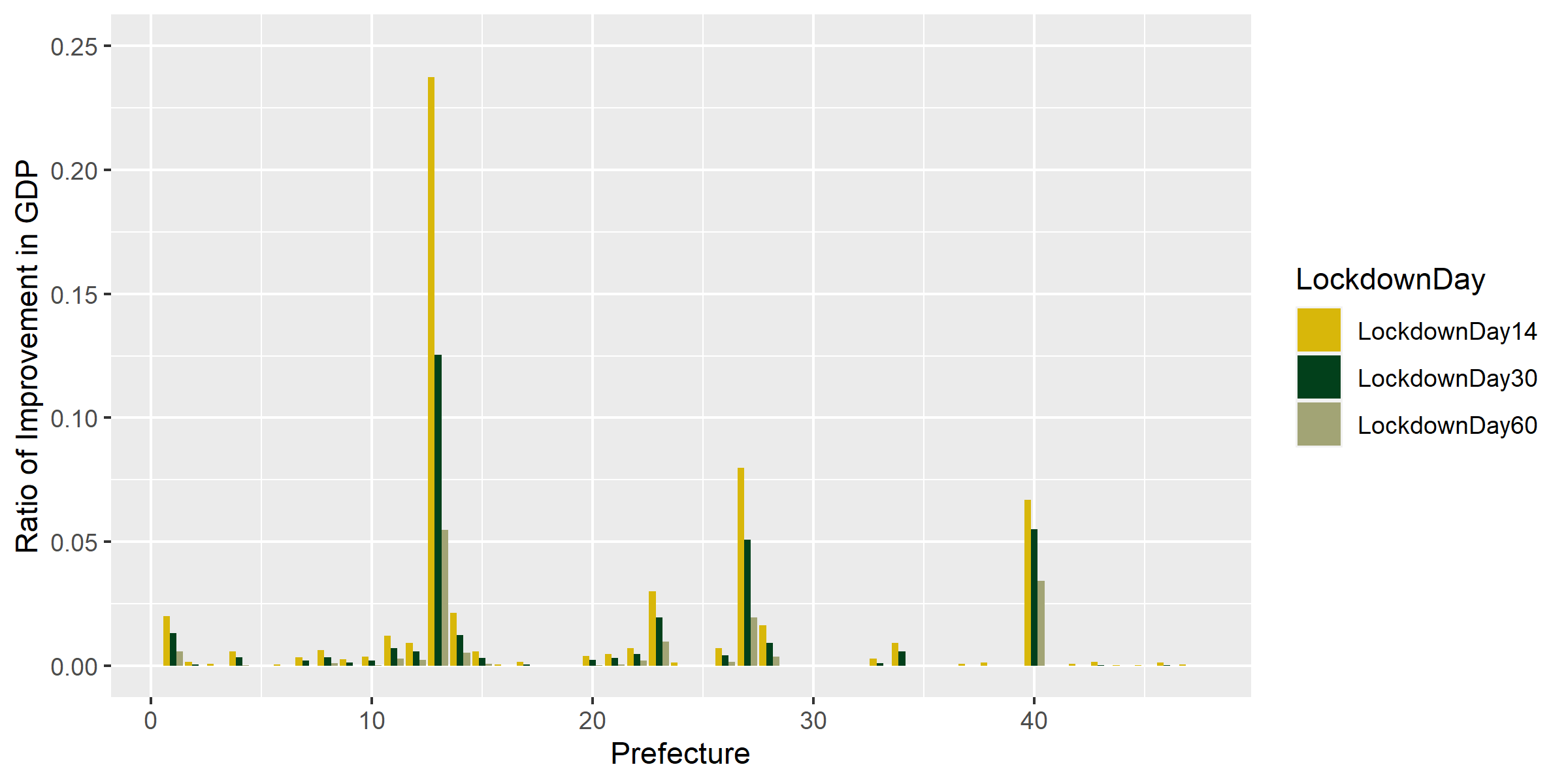

Supplement: S5 Fig — The improvement is defined as the ratio of the increase in the national GDP by each prefecture lifting its lockdown to the decrease in GDP by all prefectures’ lockdowns. The horizontal axis indicates the JIS codes of the prefectures. The yellow, dark green, and light green bars show the ratio of the improvement when lockdowns persist for 14, 30, and 60 days, respectively. (PNG) [file pone.0255031.s008.png]

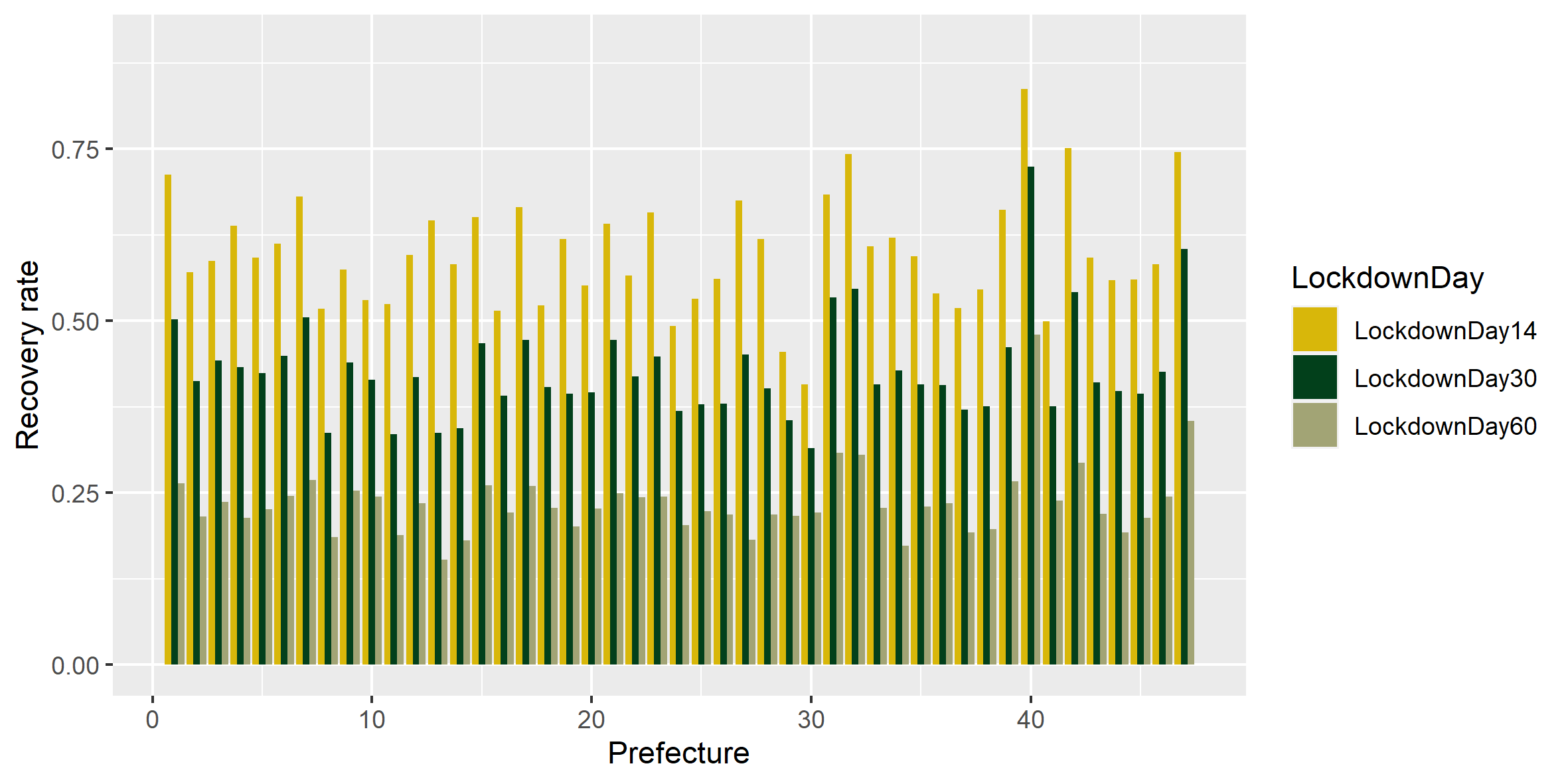

Supplement: S6 Fig — The recovery rate is defined as the ratio of the increase in the GRP of each prefecture by lifting its lockdown to the decrease in its GRP by all prefectures’ lockdowns. The horizontal axis indicates the JIS codes of the prefectures. The yellow, dark green, and light green bars show the recovery rate when lockdowns persist for 14, 30, and 60 days, respectively. (PNG) [file pone.0255031.s009.png]

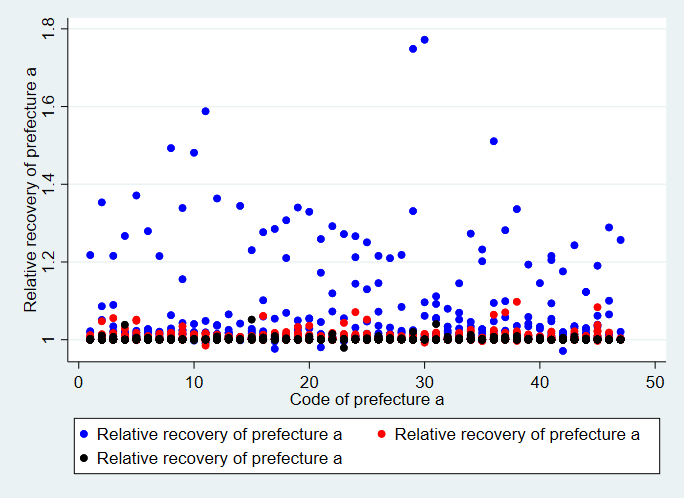

Supplement: S7 Fig — The relative recovery measure is defined as the ratio of the increase in the GRP of prefecture a when it lifts its lockdown together with prefecture b to its increase when prefecture a lifts its lockdown alone. The horizontal axis shows the JIS code of prefecture a. The colour of each dot indicates whether the GRP of prefecture b is among the top 10 (blue), the bottom 10 (black), or others (red). (PNG) [file pone.0255031.s010.png]

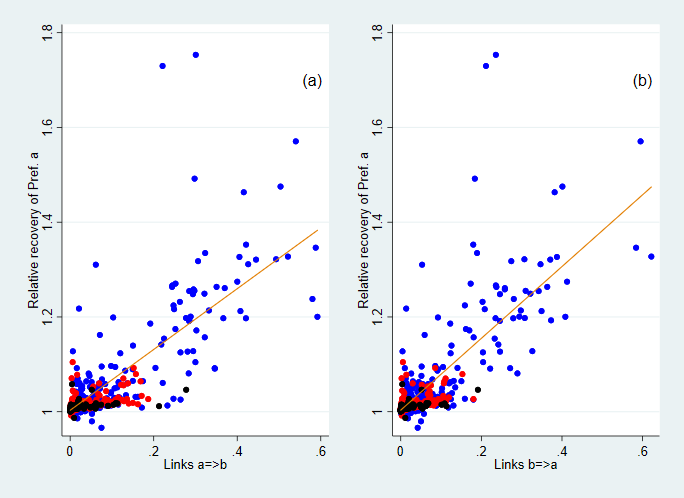

Supplement: S8 Fig — The vertical axis indicates the relative recovery of prefecture a, defined as the ratio of the increase in the GRP of prefecture a by lifting its lockdown together with prefecture b to its increase by lifting its lockdown alone. The effect of the GRP of b and total links between the two are excluded from the relative recovery measure. The variable in the horizontal axis is given by Eqs 1 and 2 in panels (a) and (b), respectively. The orange line in each panel signifies the fitted value from a linear regression that controls for the effect of the GRP of b and total number of links between a and b. The blue, black, and red dots indicate the pairs of prefectures a and b for which the GRP of b is among the top 10, bottom 10, and others, respectively. (PNG) [file pone.0255031.s011.png]

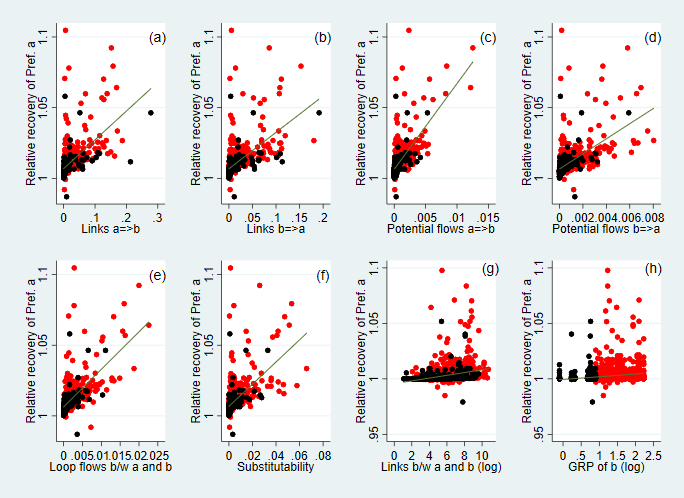

Supplement: S9 Fig — See the caption of Fig 5 and S8 Fig. for the definitions of the variables used here. The green line in each panel signifies the fitted value from a linear regression that controls for the effect of the GRP of b and total number of links between a and b in (a)–(g). The black and red dots indicate the pairs of prefectures a and b for which the GRP of b is among the bottom 10 and between 11 and 37, respectively. (PNG) [file pone.0255031.s012.png]

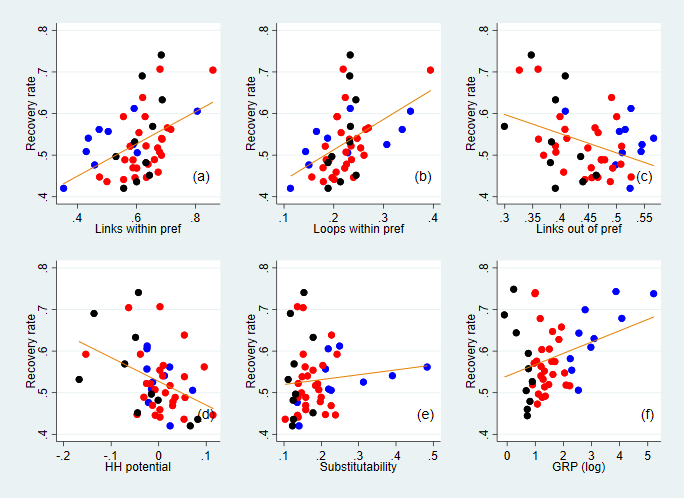

Supplement: S10 Fig — See the caption of Fig 4 for the definitions of the variables used here. The orange line in each panel specifies the fitted value from a linear regression that controls for the effect of GRP in (b)–(f). The blue, black, and red dots indicate the prefectures whose GRP is among the top 10, the bottom 10, or others, respectively. (PNG) [file pone.0255031.s013.png]

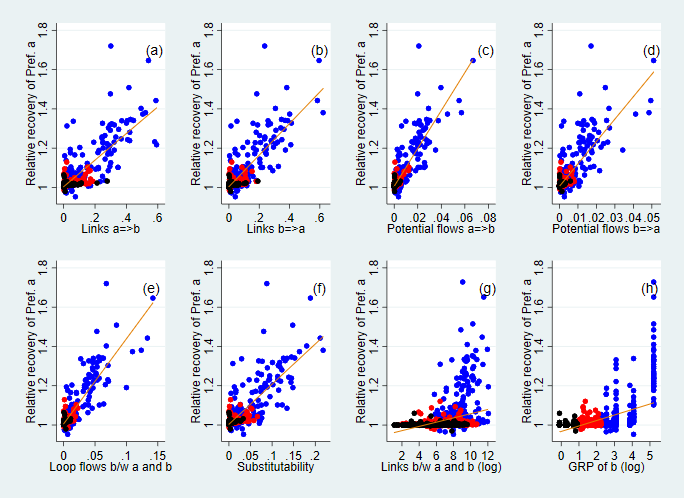

Supplement: S11 Fig — See the caption of Fig 5 for the definitions of the variables used here. The red line in each panel signifies the fitted value from a linear regression that controls for the effect of the GRP of b and total number of links between a and b in (a)–(g). The blue, black, and red dots indicate the pairs of prefectures a and b for which the GRP of b is among the top 10, the bottom 10, or others, respectively. (PNG) [file pone.0255031.s014.png]
