# Supplementary material for: Do economic effects of the anti-COVID-19 lockdowns in different regions interact through supply chains?
Source: PLoS One. 2021 Jul 30;16(7):e0255031. doi: 10.1371/journal.pone.0255031 (PMC8323942; doi:10.1371/journal.pone.0255031)
Supplement: S1 Table — Sectors are classified by the JSIC [23] at the two-digit level, except for industries 560, 561, and 569 for which we use three-digit codes to reflect the actual circumstances. The sector names are abbreviated. S1 Table lists the sector descriptions and abbreviations. (PDF) [file pone.0255031.s015.pdf]

S1 Table.. Sector-specific rates of reduction in production capacity. Sectors are classified by the JSIC [23] at the two-digit level, except for industries 560, 561, and 569 for which we use three-digit codes to reflect the actual circumstances. The sector names are abbreviated. S2 Table. lists the sector descriptions and abbreviations.

Code	Sector (abbreviated)	Reduction rate	Work-from -home rate	Exposure level	Rationale
1	AGR.	0.433	0.134	0.5	Low exposure
2	FRS.	0.433	0.134	0.5	Low exposure
3	FIS.	0.433	0.134	0.5	Low exposure
4	AQA.	0.433	0.134	0.5	Low exposure
5	MIN.	0.637	0.363	1	Ordinary
6	CNS.GEN.	0.758	0.242	1	Ordinary
7	CNS.SPC.	0.758	0.242	1	Ordinary
8	EQP.	0.758	0.242	1	Ordinary
9	MAN.FOD.	0.76	0.240	1	Ordinary
10	MAN.BEV.	0.76	0.240	1	Ordinary
11	MAN.TEX	0.668	0.332	1	Ordinary
12	MAN.LUM.	0.768	0.232	1	Ordinary
13	MAN.FUR.	0.768	0.232	1	Ordinary
14	MAN.PUL.	0.676	0.324	1	Ordinary
15	PRT.	0.676	0.324	1	Ordinary
16	MAN.CHM.	0.529	0.471	1	Ordinary
17	MAN.PET.	0.651	0.349	1	Ordinary
18	MAN.PLA.	0.704	0.296	1	Ordinary
19	MAN.RUB.	0.704	0.296	1	Ordinary
20	MAN.LET.	0.668	0.332	1	Ordinary
21	MAN.CER.	0.709	0.291	1	Ordinary
22	MAN.IRN.	0.732	0.268	1	Ordinary
23	MAN.NFM.	0.732	0.268	1	Ordinary
24	MAN.FBM.	0.695	0.305	1	Ordinary
25	MAN.GNM.	0.604	0.396	1	Ordinary
26	MAN.PRM.	0.604	0.396	1	Ordinary
27	MAN.BSM.	0.604	0.396	1	Ordinary
28	EPT.	0.333	0.667	1	Ordinary
29	MAN.ELM.	0.58	0.420	1	Ordinary
30	MAN.INF.	0.333	0.667	1	Ordinary
31	MAN.TRN.	0.504	0.496	1	Ordinary
32	MAN.MSC.	0.705	0.295	1	Ordinary
33	ELE.	0.0623	0.377	0.1	Lifeline
34	GAS.	0.0623	0.377	0.1	Lifeline
35	HET.	0.0623	0.377	0.1	Lifeline
36	WTR.	0.0623	0.377	0.1	Lifeline
37	COM.	0.0401	0.599	0.1	Lifeline
38	BRD.	0.0192	0.808	0.1	Lifeline
39	INF.SVC.	0.097	0.903	1	Ordinary
40	INT.	0.0401	0.599	0.1	Lifeline
41	INF.DST.	0.192	0.808	1	Ordinary
42	RLW.TRP.	0.0701	0.299	0.1	Lifeline
43	PAS.TRP.	0.0701	0.299	0.1	Lifeline
44	FRE.TRP.	0.0701	0.299	0.1	Lifeline
45	WTR.TRP.	0.0701	0.299	0.1	Lifeline
46	AIR.TRP.	0.0701	0.299	0.1	Lifeline
47	WRH.	0.0701	0.299	0.1	Lifeline
48	SVC.TRP.	0.0701	0.299	0.1	Lifeline
49	PST.SVC.	0.0701	0.299	0.1	Lifeline
50	WHL.GEN.	0.525	0.475	1	Ordinary
51	WHL.TEX.	0.525	0.475	1	Ordinary
52	WHL.FOD.	0.525	0.475	1	Ordinary
53	WHL.MAT.	0.525	0.475	1	Ordinary
54	WHL.MCN.	0.525	0.475	1	Ordinary
55	WHL.MSC.	0.525	0.475	1	Ordinary
560	RTL.ADM.	0.525	0.475	1	Ordinary
561	RTL.DPT.	0.525	0.475	1	Closed
569	RTL.GNM.	0.0525	0.475	0.1	Lifeline
57	RTL.GEN.	0.525	0.475	1	Ordinary

58	RTL.FOD.	0.525	0.475	1	Ordinary
59	RTL.MCN.	0.525	0.475	1	Ordinary
60	RTL.MSC.	0.525	0.475	1	Ordinary
61	RTL.NST.	0.525	0.475	1	Ordinary
62	FIN.BNK.	0.214	0.786	1	Ordinary
63	FIN.ORG.	0.214	0.786	1	Ordinary
64	FIN.LON.	0.214	0.786	1	Ordinary
65	FIN.TRN.	0.214	0.786	1	Ordinary
66	FIN.AUX.	0.214	0.786	1	Ordinary
67	INS.	0.214	0.786	1	Ordinary
68	RST.AGN.	0.423	0.577	1	Ordinary
69	RTS.LES.	0.423	0.577	1	Ordinary
70	RNT.	0.362	0.638	1	Ordinary
71	SCI.	0.172	0.828	1	Ordinary
72	SVC.PRF.	0.362	0.638	1	Ordinary
73	ADV.	0.362	0.638	1	Ordinary
74	SVC.TEC.	0.362	0.638	1	Ordinary
75	ACM.	0.889	0.111	1	Closed
76	EAT.	0.889	0.111	1	Ordinary
77	DEL.	0.0521	0.479	0.1	Lifeline
78	LND.	0.521	0.479	1	Ordinary
79	SVC.PSN.	0.521	0.479	1	Ordinary
80	SVC.AMS.	0.521	0.479	1	Closed
81	SCH.	0.086	0.828	0.5	Low exposure
82	EDC.	0.086	0.828	0.5	Low exposure
83	MED.	0.0753	0.247	0.1	Lifeline
84	HLT.	0	0.247	0	Sustantial
85	WEL.	0	0.247	0	Sustantial
86	PST.OFC.	0.0362	0.638	0.1	Lifeline
87	CAS.	0.181	0.638	0.5	Low exposure
88	WAS.	0.181	0.638	0.5	Low exposure
89	SVC.AUT.	0.181	0.638	0.5	Low exposure
90	SVC.MCN.	0.181	0.638	0.5	Low exposure
91	SVC.EMP.	0.181	0.638	0.5	Low exposure
92	SVC.BUS.	0.181	0.638	0.5	Low exposure
93	PLT.	0.181	0.638	0.5	Low exposure
94	REL.	0.181	0.638	0.5	Low exposure
95	SVC.MSC.	0.181	0.638	0.5	Low exposure
96	GOV.INT.	0.0515	0.485	0.1	Lifeline
97	NA	0.0515	0.485	0.1	Lifeline
98	GOV.LOC.	0.0515	0.485	0.1	Lifeline
99	NEC	0.362	0.638	1	Ordinary
