# Supplementary material for: Do economic effects of the anti-COVID-19 lockdowns in different regions interact through supply chains?
Source: PLoS One. 2021 Jul 30;16(7):e0255031. doi: 10.1371/journal.pone.0255031 (PMC8323942; doi:10.1371/journal.pone.0255031)
Supplement: S2 Table — (PDF) [file pone.0255031.s016.pdf]

S2 Table.. Sector classifications and abbreviations.

Code	Description	Abbreviation
01	AGRICULTURE	AGR.
02	FORESTRY	FRS.
03	FISHERIES, EXCEPT AQUACULTURE	FIS.
04	AQUACULTURE	AQA.
05	MINING AND QUARRYING OF STONE AND GRAVEL	MIN.
06	CONSTRUCTION WORK, GENERAL INCLUDING PUBLIC AND PRIVATE CONSTRUCTION WORK	CNS.GEN.
07	CONSTRUCTION WORK BY SPECIALIST CONTRACTOR, EXCEPT EQUIPMENT INSTALLATION WORK	CNS.SPC.
08	EQUIPMENT INSTALLATION WORK	EQP.
09	MANUFACTURE OF FOOD	MAN.FOD.
10	MANUFACTURE OF BEVERAGES, TOBACCO AND FEED	MAN.BEV.
11	MANUFACTURE OF TEXTILE PRODUCTS	MAN.TEX
12	MANUFACTURE OF LUMBER AND WOOD PRODUCTS, EXCEPT FURNITURE	MAN.LUM.
13	MANUFACTURE OF FURNITURE AND FIXTURES	MAN.FUR.
14	MANUFACTURE OF PULP, PAPER AND PAPER PRODUCTS	MAN.PUL.
15	PRINTING AND ALLIED INDUSTRIES	PRT.
16	MANUFACTURE OF CHEMICAL AND ALLIED PRODUCTS	MAN.CHM.
17	MANUFACTURE OF PETROLEUM AND COAL PRODUCTS	MAN.PET.
18	MANUFACTURE OF PLASTIC PRODUCTS, EXCEPT OTHERWISE CLASSIFIED	MAN.PLA.
19	MANUFACTURE OF RUBBER PRODUCTS	MAN.RUB.
20	MANUFACTURE OF LEATHER TANNING, LEATHER PRODUCTS AND FUR SKINS	MAN.LET.
21	MANUFACTURE OF CERAMIC, STONE AND CLAY PRODUCTS	MAN.CER.
22	MANUFACTURE OF IRON AND STEEL	MAN.IRN.
23	MANUFACTURE OF NON-FERROUS METALS AND PRODUCTS	MAN.NFM.
24	MANUFACTURE OF FABRICATED METAL PRODUCTS	MAN.FBM.
25	MANUFACTURE OF GENERAL-PURPOSE MACHINERY	MAN.GNM.
26	MANUFACTURE OF PRODUCTION MACHINERY	MAN.PRM.
27	MANUFACTURE OF BUSINESS ORIENTED MACHINERY	MAN.BSM.
28	ELECTRONIC PARTS, DEVICES AND ELECTRONIC CIRCUITS	EPT.
29	MANUFACTURE OF ELECTRICAL MACHINERY, EQUIPMENT AND SUPPLIES	MAN.ELM.
30	MANUFACTURE OF INFORMATION AND COMMUNICATION ELECTRONICS EQUIPMENT	MAN.INF.
31	MANUFACTURE OF TRANSPORTATION EQUIPMENT	MAN.TRN.
32	MISCELLANEOUS MANUFACTURING INDUSTRIES	MAN.MSC.
33	PRODUCTION, TRANSMISSION AND DISTRIBUTION OF ELECTRICITY	ELE.
34	PRODUCTION AND DISTRIBUTION OF GAS	GAS.
35	HEAT SUPPLY	HET.
36	COLLECTION, PURIFICATION AND DISTRIBUTION OF WATER, AND SEWAGE COLLECTION, PROCESSING	WTR.
37	COMMUNICATIONS	COM.

38	BROADCASTING	BRD.
39	INFORMATION SERVICES	INF.SVC.
40	SERVICES INCIDENTAL TO INTERNET	INT.
41	VIDEO PICTURE INFORMATION, SOUND INFORMATION, CHARACTER INFORMATION PRODUCTION AND DISTRIBUTION	INF.DST.
42	RAILWAY TRANSPORT	RLW.TRP.
43	ROAD PASSENGER TRANSPORT	PAS.TRP.
44	ROAD FREIGHT TRANSPORT	FRE.TRP.
45	WATER TRANSPORT	WTR.TRP.
46	AIR TRANSPORT	AIR.TRP.
47	WAREHOUSING	WRH.
48	SERVICES INCIDENTAL TO TRANSPORT	SVC.TRP.
49	POSTAL SERVICES, INCLUDING MAIL DELIVERY	PST.SVC.
50	WHOLESALE TRADE, GENERAL MERCHANDISE	WHL.GEN.
51	WHOLESALE TRADE (TEXTILE AND APPAREL)	WHL.TEX.
52	WHOLESALE TRADE (FOOD AND BEVERAGES)	WHL.FOD.
53	WHOLESALE TRADE (BUILDING MATERIALS, MINERALS AND METALS, ETC)	WHL.MAT.
54	WHOLESALE TRADE (MACHINERY AND EQUIPMENT)	WHL.MCN.
55	MISCELLANEOUS WHOLESALE TRADE	WHL.MSC.
560	ESTABLISHMENTS ENGAGED IN ADMINISTRATIVE OR ANCILLARY ECONOMIC ACTIVITIES	RTL.ADM.
561	DEPARTMENT STORES AND GENERAL MERCHANDISE SUPERMARKET	RTL.DPT.
569	MISCELLANEOUS RETAIL TRADE, GENERAL MERCHANDISE	RTL.GNM.
57	RETAIL TRADE, GENERAL MERCHANDISE	RTL.GEN.
58	RETAIL TRADE (FOOD AND BEVERAGE)	RTL.FOD.
59	RETAIL TRADE (MACHINERY AND EQUIPMENT)	RTL.MCN.
60	MISCELLANEOUS RETAIL TRADE	RTL.MSC.
61	NONSTORE RETAILERS	RTL.NST.
62	BANKING	FIN.BNK.
63	FINANCIAL INSTITUTIONS FOR COOPERATIVE ORGANIZATIONS	FIN.ORG.
64	NON-DEPOSIT MONEY CORPORATIONS, INCLUDING LENDING AND CREDIT CARD BUSINESS	FIN.LON.
65	FINANCIAL PRODUCTS TRANSACTION DEALERS AND FUTURES COMMODITY TRANSACTION DEALERS	FIN.TRN.
66	FINANCIAL AUXILIARIES	FIN.AUX.
67	INSURANCE INSTITUTIONS, INCLUDING INSURANCE AGENTS, BROKERS AND SERVICES	INS.
68	REAL ESTATE AGENCIES	RST.AGN.
69	REAL ESTATE LESSORS AND MANAGERS	RTS.LES.
70	GOODS RENTAL AND LEASING	RNT.
71	SCIENTIFIC AND DEVELOPMENT RESEARCH INSTITUTES	SCI.
72	PROFESSIONAL SERVICES, N.E.C.	SVC.PRF.
73	ADVERTISING	ADV.
74	TECHNICAL SERVICES, N.E.C.	SVC.TEC.

75	ACCOMMODATION	ACM.
76	EATING AND DRINKING PLACES	EAT.
77	FOOD TAKE OUT AND DELIVERY SERVICES	DEL.
78	LAUNDRY, BEAUTY AND BATH SERVICES	LND.
79	MISCELLANEOUS LIVING-RELATED AND PERSONAL SERVICES	SVC.PSN.
80	SERVICES FOR AMUSEMENT AND RECREATION	SVC.AMS.
81	SCHOOL EDUCATION	SCH.
82	MISCELLANEOUS EDUCATION, LEARNING SUPPORT	EDC.
83	MEDICAL AND OTHER HEALTH SERVICE	MED.
84	PUBLIC HEALTH AND HYGIENE	HLT.
85	SOCIAL INSURANCE, SOCIAL WELFARE AND CARE SERVICES	WEL.
86	POSTAL OFFICE	PST.OFC.
87	COOPERATIVE ASSOCIATIONS, N.E.C.	CAS.
88	WASTE DISPOSAL BUSINESS	WAS.
89	AUTOMOBILE MAINTENANCE SERVICES	SVC.AUT.
90	MACHINE, ETC. REPAIR SERVICES, EXCEPT OTHERWISE CLASSIFIED	SVC.MCN.
91	EMPLOYMENT AND WORKER DISPATCHING SERVICES	SVC.EMP.
92	MISCELLANEOUS BUSINESS SERVICES	SVC.BUS.
93	POLITICAL, BUSINESS AND CULTURAL ORGANIZATIONS	PLT.
94	RELIGION	REL.
95	MISCELLANEOUS SERVICES	SVC.MSC.
96	FOREIGN GOVERNMENTS AND INTERNATIONAL AGENCIES IN JAPAN	GOV.INT.
97	NATIONAL GOVERNMENT SERVICES	GOV.NAT.
98	LOCAL GOVERNMENT SERVICES	GOV.LOC.
99	INDUSTRIES UNABLE TO CLASSIFY	NEC
