# Supplementary material for: Do economic effects of the anti-COVID-19 lockdowns in different regions interact through supply chains?
Source: PLoS One. 2021 Jul 30;16(7):e0255031. doi: 10.1371/journal.pone.0255031 (PMC8323942; doi:10.1371/journal.pone.0255031)
Supplement: S3 Table — The definitions of the variables are as follows. RecRatio: the recovery rate defined as the ratio of the increase in the GRP of each prefecture by lifting its lockdown to the decrease in its GRP by all prefectures’ lockdowns. GRP: gross regional product (log). Links: the degree (log). InLink: the share of links within the prefecture to all its links. InLoop: the share of loop flows within the prefecture to all its flows. OutLink: the share of outward inter-prefectural links to all the links of the prefecture. Potential: the average HH potential of the firms in the prefecture. Sub: the share of substitutable suppliers to all suppliers of the prefecture located outside the prefecture. (PDF) [file pone.0255031.s017.pdf]

S3 Table.. Correlation matrix of the variables used in Section 4.3. The definitions of the variables are as follows. RecRatio: the recovery rate defined as the ratio of the increase in the GRP of each prefecture by lifting its lockdown to the decrease in its GRP by all prefectures' lockdowns. GRP: gross regional product (log). Links: the degree (log). InLink: the share of links within the prefecture to all its links. InLoop: the share of loop flows within the prefecture to all its flows. OutLink: the share of outward inter-prefectural links to all the links of the prefecture. Potential: the average HH potential of the firms in the prefecture. Sub: the share of substitutable suppliers to all suppliers of the prefecture located outside the prefecture.

Variable	RecRatio	GRP	Degree	InLink	InLoop	OutLink	Potential	Sub
RecRatio	1.000							
GRP	0.311	1.000						
Degree	0.370	0.965	1.000					
InLink	0.218	-0.467	-0.374	1.000				
InLoop	0.432	0.072	0.151	0.720	1.000			
OutLink	-0.046	0.676	0.661	-0.688	-0.351	1.000		
Potential	-0.321	0.104	0.090	-0.046	-0.076	0.193	1.000	
Sub	0.449	0.803	0.829	-0.246	0.307	0.573	0.096	1.000
