# Supplementary material for: Do economic effects of the anti-COVID-19 lockdowns in different regions interact through supply chains?
Source: PLoS One. 2021 Jul 30;16(7):e0255031. doi: 10.1371/journal.pone.0255031 (PMC8323942; doi:10.1371/journal.pone.0255031)
Supplement: S5 Table — The definitions of the variables are as follows. Recova: the relative recovery of prefecture a defined as the ratio of the increase in the GRP of prefecture a by lifting its lockdown together with prefecture b to its increase by lifting its lockdown alone. Linkab: the share of links from a to b to all links from a. Linkba: the share of links from b to a to all links from a. Potab: the share of potential flows from b to a to the total links of a. Potba: the share of potential flows from a to b to the total links of a. Subab: the share of suppliers substitutable by those in b to a’s suppliers outside a and b. Subba: the share of suppliers substitutable by those in a to b’s suppliers outside a and b. Loopab: the share of loop flows between a and b to the total flows between the two. Biab: the number of inter-prefecture links between a and b in logs. GRPj: GRP of b in logs. (PDF) [file pone.0255031.s019.pdf]

S5 Table.. Correlation matrix of the variables used in Section 4.4. The definitions of the variables are as follows. $Recov_a$: the relative recovery of prefecture a defined as the ratio of the increase in the GRP of prefecture a by lifting its lockdown together with prefecture b to its increase by lifting its lockdown alone. $Link_{ab}$: the share of links from a to b to all links from a. $Link_{ba}$: the share of links from b to a to all links from a. Pot_{ab}: the share of potential flows from b to a to the total links of a. Pot_{ba}: the share of potential flows from a to b to the total links of a. Sub_{ab}: the share of suppliers substitutable by those in b to a's suppliers outside a and b. Sub_{ba}: the share of suppliers substitutable by those in a to b's suppliers outside a and b. $Loop_{ab}$: the share of loop flows between a and b to the total flows between the two. Bi_{ab}: the number of inter-prefecture links between a and b in logs. GRP_j: GRP of b in logs.

Variable	$Recov_a$	$Link_{ab}$	$Link_{ba}$	Pot_{ab}	Pot_{ba}	Sub_{ab}	Sub_{ba}	$Loop_{ab}$	Bi_{ab}	[illegible]
$Recov_a$	1.000									
$Link_{ab}$	0.820	1.000								
$Link_{ba}$	0.818	0.966	1.000							
Pot_{ab}	0.870	0.927	0.961	1.000						
Pot_{ba}	0.808	0.915	0.955	0.968	1.000					
Sub_{ab}	0.071	0.185	0.238	0.182	0.243	1.000				
Sub_{ba}	0.813	0.961	0.966	0.946	0.948	0.237	1.000			
$Loop_{ab}$	0.879	0.911	0.952	0.986	0.979	0.206	0.940	1.000		
Bi_{ab}	0.392	0.543	0.564	0.499	0.528	0.572	0.572	0.504	1.000	
GRP_b	0.563	0.610	0.597	0.602	0.582	0.056	0.643	0.596	0.576	1.[illegible]
