# Supplementary material for: Do economic effects of the anti-COVID-19 lockdowns in different regions interact through supply chains?
Source: PLoS One. 2021 Jul 30;16(7):e0255031. doi: 10.1371/journal.pone.0255031 (PMC8323942; doi:10.1371/journal.pone.0255031)
Supplement: S6 Table — The dependent variable is the relative recovery measure. See the caption of Table S5 Table for the definitions of the independent variables. Standard errors are in parentheses. *** p<0.01, ** p<0.05, * p<0.1. (PDF) [file pone.0255031.s020.pdf]

S6 Table.. Regression results for Section 4.4. The dependent variable is the relative recovery measure. See the caption of Table S5 Table. for the definitions of the independent variables. Standard errors are in parentheses. *** p<0.01, ** p<0.05, * p<0.1.

	(1)	(2)	(3)	(4)	(5)	(6)	(7)
$Link_{ab}$	0.519***						0.440***
	(0.0175)						(0.0306)
$Link_{ba}$		0.619***					-0.375***
		(0.0199)					(0.0460)
Pot_{ab}			8.333***				0.277
			(0.198)				(0.624)
Pot_{ba}				8.076***			-17.82***
				(0.271)			(0.644)
$Loop_{ab}$					3.841***		10.06***
					(0.0844)		(0.309)
Sub_{ba}						1.564***	-0.248**
						(0.0550)	(0.0989)
Bi_{ab}	-0.00211***	-0.00288***	-0.00182***	-0.00174***	-0.00225***	-0.00235***	-0.000602**
	(0.000413)	(0.000415)	(0.000352)	(0.000407)	(0.000341)	(0.000423)	(0.000298)
GRP_b	-0.0186***	-0.0186***	-0.0135***	-0.0232***	-0.0120***	-0.0220***	-0.00540***
	(0.00210)	(0.00205)	(0.00181)	(0.00202)	(0.00175)	(0.00208)	(0.00146)
GRP_b^2	0.00652***	0.00676***	0.00467***	0.00795***	0.00431***	0.00712***	0.00192***
	(0.000490)	(0.000471)	(0.000422)	(0.000458)	(0.000405)	(0.000489)	(0.000350)
Constant	1.019***	1.023***	1.016***	1.021***	1.017***	1.024***	1.006***
	(0.00236)	(0.00233)	(0.00208)	(0.00236)	(0.00200)	(0.00239)	(0.00168)
Observations	2,162	2,162	2,162	2,162	2,162	2,162	2,162
R-squared	0.713	0.721	0.778	0.714	0.794	0.706	0.865
